# Whole-Exome Sequencing and cfDNA Analysis Uncover Genetic Determinants of Melanoma Therapy Response in a Real-World Setting

**DOI:** 10.3390/ijms24054302

**Published:** 2023-02-21

**Authors:** Irene Vanni, Lorenza Pastorino, Enrica Teresa Tanda, Virginia Andreotti, Bruna Dalmasso, Nicola Solari, Matteo Mascherini, Francesco Cabiddu, Antonio Guadagno, Simona Coco, Eleonora Allavena, William Bruno, Gabriella Pietra, Michela Croce, Rosaria Gangemi, Michele Piana, Gabriele Zoppoli, Lorenzo Ferrando, Francesco Spagnolo, Paola Queirolo, Paola Ghiorzo

**Affiliations:** 1 Genetics of Rare Cancers, IRCCS Ospedale Policlinico San Martino, 16132 Genoa, Italy; 2 Department of Internal Medicine and Medical Specialties (DiMI), University of Genoa, 16132 Genoa, Italy; 3 Medical Oncology 2, IRCCS Ospedale Policlinico San Martino, 16132 Genoa, Italy; 4 Surgical Oncology, IRCCS Ospedale Policlinico San Martino, 16132 Genoa, Italy; 5 Surgical Clinic Unit 1, IRCCS Ospedale Policlinico San Martino, 16132 Genoa, Italy; 6 Anatomic Pathology Unit, IRCCS Ospedale Policlinico San Martino, 16132 Genoa, Italy; 7 Lung Cancer Unit, IRCCS Ospedale Policlinico San Martino, 16132 Genoa, Italy; 8 IRCCS Ospedale Policlinico San Martino, U.O. Immunologia, 16132 Genoa, Italy; 9 Department of Experimental Medicine (DiMES), University of Genoa, 16132 Genoa, Italy; 10 Bioterapie, IRCCS Ospedale Policlinico San Martino, 16132 Genoa, Italy; 11 Dipartimento di Matematica (MIDA), University of Genoa, 16132 Genoa, Italy; 12 Life Science Computational Laboratory (LISCOMP), IRCCS Ospedale Policlinico San Martino, 16132 Genoa, Italy; 13 Clinica di Medicina Interna a Indirizzo Oncologico, IRCCS Ospedale Policlinico San Martino, 16132 Genoa, Italy; 14 Dipartimento di Scienze Chirurgiche e Diagnostiche Integrate (DISC), University of Genoa, 16132 Genoa, Italy; 15 Melanoma, Sarcoma & Rare Tumors Division, European Institute of Oncology (IEO), 20141 Milan, Italy

**Keywords:** melanoma, whole-exome sequencing, circulating free DNA, *BRAF* V600, targeted therapy, immunotherapy, germline pathogenic variants, tumor ploidy, tumor mutational burden, loss of heterozygosity

## Abstract

Although several studies have explored the molecular landscape of metastatic melanoma, the genetic determinants of therapy resistance are still largely unknown. Here, we aimed to determine the contribution of whole-exome sequencing and circulating free DNA (cfDNA) analysis in predicting response to therapy in a consecutive real-world cohort of 36 patients, undergoing fresh tissue biopsy and followed during treatment. Although the underpowered sample size limited statistical analysis, samples from non-responders had higher copy number variations and mutations in melanoma driver genes compared to responders in the *BRAF* V600+ subset. In the *BRAF* V600− subset, Tumor Mutational Burden (TMB) was twice that in responders vs. non-responders. Genomic layout revealed commonly known and novel potential intrinsic/acquired resistance driver gene variants. Among these, *RAC1*, *FBXW7*, *GNAQ* mutations, and *BRAF*/*PTEN* amplification/deletion were present in 42% and 67% of patients, respectively. Both Loss of Heterozygosity (LOH) load and tumor ploidy were inversely associated with TMB. In immunotherapy-treated patients, samples from responders showed higher TMB and lower LOH and were more frequently diploid compared to non-responders. Secondary germline testing and cfDNA analysis proved their efficacy in finding germline predisposing variants carriers (8.3%) and following dynamic changes during treatment as a surrogate of tissue biopsy, respectively.

## 1. Introduction

Melanoma is one of the most aggressive malignancies of the skin. Its incidence and prevalence are growing globally, partly because of the increase in early diagnoses [1,2]. Until 10 years ago, advanced melanoma resulted in poor survival due to the lack of durable responses to conventional therapy [3], with a median overall survival of about 6 months in stage IV melanoma patients. Since 2011, however, the rules of stage IV melanoma treatment have been completely rewritten, with the introduction of Targeted Therapies (TTs) with *BRAF* and MEK inhibitors (*BRAF*+*MEKi*) [4,5,6], and of immunotherapy with anti CTLA-4 [7] and anti-*PD-1* [8,9]. Monotherapy and combined therapy with these new agents has improved melanoma prognosis, resulting in a 5-year survival rate of 34–43% and a 6-and-a-half-year survival rate of nearly 50%, respectively [10,11,12]. However, mainly because of primary and acquired resistance to treatments, most individuals ultimately relapse. Moreover, only patients with *BRAF*-mutant tumors, which account for about 50% of all cutaneous melanoma, are eligible for TT with *BRAF*+*MEKi* [13]. The current therapeutic scenario for patients with *BRAF*-mutant melanoma has been extensively described [14]. Several preclinical and clinical trials are investigating potentially actionable molecules and pathways to tackle multiple resistance mechanisms simultaneously. The advent of massive parallel sequencing, allowing the simultaneous analysis of several genes has led, in the past two decades, to Whole-Exome Sequencing (WES) and Whole-Genome Sequencing (WGS) studies that allowed the identification of several potential therapeutic targets. Since the discovery of the first actionable mutation (*BRAF* V600), several other putative drivers of melanomagenesis and/or melanoma progression have been identified, and others are currently being assessed, prompting pharmacogenomics studies on potentially actionable targets [15]. However, melanoma is among the tumors with the highest mutation burden, and results from different studies are frequently not overlapping, possibly due to dissimilar sample sizes and cohort characteristics [16,17,18,19,20]. Although high mutational burden is one of the reasons behind the success of immunotherapy in this tumor, it hampers the identification of novel potentially actionable driver genes [21]. The mutational landscape of non-*BRAF* skin melanoma, in light of recent data deriving from WES or WGS studies, includes 33 candidate driver genes altered with a frequency greater than 1.5% [22]. The complex scenario of driver mutations and Copy Number Variations (CNVs) identified has changed the paradigm of melanoma pathogenesis. Large gene panels will be increasingly used in clinical practice for the molecular classification of most human cancers, simplifying the methodology (including data interpretation) and reducing the overall costs.

However, extensive molecular characterization, potentially resulting in additional targets for new drugs or predictive/prognostic biomarkers, creates possibilities for the adoption of exome/genome level sequencing in real-world melanoma clinical settings.

In this context, the analysis of circulating free DNA (cfDNA) has recently emerged as a valid biological tool for non-invasive and quantitative characterization of the whole tumor genome, as well as for identification of tumor heterogeneity, drug resistance, and clonal evolution during treatment and disease progression [23].

This study focused on better understanding the correlation between the genetic profile and systemic treatment with TT or Immune Checkpoint Inhibitor (ICI) agents in a real-world series of melanoma patients consecutively enrolled and classified as Responders (R) or Non-Responders (NR) in order to pave the way to discriminate patients who could benefit from one treatment over another. 

## 2. Results

### 2.1. BRAF Status in Tumor Samples Associated with Clinical Response 

Thirty-six tumor samples from 19 *BRAF* V600+ and 17 *BRAF* V600− patients (three of whom had pathogenetic mutations in the *KIT* gene) were analyzed and their mutational profiles/CNV/LOH correlated with clinical response. Overall, 17 patients were considered Responders (R) and 19 Non-Responders (NR); we used pre-therapy and post-therapy tumor samples from 13 and 23 patients, respectively. Eight *BRAF* V600+ tumors were from patients responding to TT or ICI agents, while 11 tumors were from patients non-responding to TT, ICI agents, and adjuvant TT. Nine *BRAF* V600− tumors were from patients responding to ICI agents and eight were from NR patients (Supplementary Table S1).

### 2.2. Genetic Layout Associated with Response to Therapy

*BRAF* V600 mutations were identified in 52.8% of patients’ tumor samples (19/36), RAS mutations in 30.6% (11/36), and non-*BRAF*/non-*NRAS* mutations in 19.4% (7/36). In one sample, #56, *BRAF* and *NRAS* were found mutated. Among the six non-*BRAF*/non-*NRAS* mutant samples, one (#34) had an *NF1* mutation (NM_001042492.3: c.3089C>T, p.Ser1030Leu) with an Allele Frequency (AF) of 10.3%. Two other samples (#14 and #21) showed co-occurrence of *NF1* mutation with a *BRAF* or *NRAS* mutation, respectively. Specifically, sample #21 harbored an *NF1* mutation (NM_001042492.3: c.6278C>, p.Ser2093Phe, AF of 30%) coexisting with the *NRAS* p.Gln61Arg mutation. Moreover, sample #14 had two different *NF1* mutations coexisting with *BRAF* p.Val600Glu. In one sample (#56), the lesion resected after therapy showed an *NRAS* mutation, but lacked the *BRAF* V600 mutation detected in the pre-therapy bioptic sample; based on the pre-therapy sample, this patient was considered to have a *BRAF* V600 mutant when choosing the adjuvant therapy regimen (Supplementary Figure S1). In all samples, *BRAF* V600+ mutations found by WES were validated by *BRAF* Multiplex Ligation-dependent Probe Amplification (MLPA) analysis and/or Sanger sequencing, confirming data from Formalin-Fixed Paraffin-Embedded (FFPE) samples analyzed for diagnostic purposes.

The number of melanoma driver gene mutations did not significantly differ according to response to therapy, although in the *BRAF* V600+ subset, the median number of melanoma driver gene mutations in NR was twice that in R (Supplementary Table S2). In contrast, no differences for both driver melanoma genes and interferon pathway genes were found in the *BRAF* V600− subset.

*TERT* promoter sequencing showed activating mutations (C228T and/or C250T) in 73.3% of the 30 tumor samples analyzed. Interestingly, in 16 *BRAF* V600+ samples for which *TERT* mutational status was available, all R and 70% NR had these mutations. Conversely, no difference was observed in the 14 *BRAF* V600− analyzed samples.

Median mutational tumor load did not significantly differ in samples from R compared to NR patients. However, in the *BRAF* V600− subset, median TMB of responding was twice that of NR (Supplementary Table S2 and Supplementary Figure S2).

CNV analysis of melanoma driver genes showed that median CNV frequency was 2.6 times higher in *BRAF* V600+ NR than in *BRAF* V600+ R samples. No difference was found in the *BRAF* V600− subset (Supplementary Table S2 and Supplementary Figure S3). The overall frequency of CNVs did not differ according to response to therapy. When looking at the *BRAF* V600+ subset, however, the number of driver genes with at least one CNV event was higher, albeit not significantly, in NR than in R. Interestingly, the most frequent melanoma driver genes showing amplifications were *HRAS* (11p15.5), *GNA11* (19p13.3), *STK19* (6p21.33), *MAP2K2* (19p13.3), *EZH2* (7q36.1), *TERT* (5p15.33), and *MTOR* (1p36.22), whereas the most frequently deleted region was located in the chromosome 10 cytoband 10q23.31 containing the *PTEN* gene. When looking at *HLA-A*, *HLA-B*, *HLA-C*, *TAP2*, *PD-1* (PDCD1), *PD-L1* (CD274), and *PDL2* (PDCD1LG2) genes, as possible determinants of response in 17 *BRAF* V600− patients, we found at least one amplification in one of these genes in six samples from six patients, four of whom (66%) were R. 

LOH analysis was evaluated in DNA Damage Repair (DDR) genes. The median number of genes with LOH was higher in NR compared to R both in *BRAF* V600+ and V600− subsets (Supplementary Tables S2 and S3).

Interestingly, the agreement on 36 tumors among the three tools (DeconstructSigs, SigMa, and SigProfiler v3.2) used to identify mutational signatures was 94.4% (Supplementary Table S4). Only two tumor samples, #55 and #43, showed discordance between DeconstructSigs/SigMa and SigProfiler (Signature_clock-like vs. SBS7 for #55; Signature_msi vs. SBS1 for #43). COSMIC mutational signatures calculated by SigProfiler v.3.2 showed SBS3, SBS5, and SBS7 as the three predominant signatures, with no association with response to therapy. Conversely, signature SBS5 was more represented in the NR cohort (26.3% vs. 17.6%) (Supplementary Table S4). The SBS5 is clock-like, as the number of mutations correlates with the individual’s age. The accumulation of SBS5 mutations over time varies across cancer types, but the cause underlying SBS5 mutations is unknown and likely reflects a collective of endogenous background mutational processes. In our cohort, five NR patients displayed the SBS5, with median age at tumor diagnosis of 63, not correlating with a higher TMB median (3.8). In the subset of R samples bearing the SBS5 signature, median age was 57 years, and TMB median was 112.4. Overall, the most prevalent SBS signature found in our study cohort was SBS7 (16/36; 44.4%), a signature linked to UV-radiation.

None of these comparisons were statistically significant, as shown in Supplementary Table S2.

### 2.3. Loss of Heterozygosity (LOH) Load and Tumor Ploidy Inversely Associated with Tumor Mutational Burden (TMB)

We observed a positive association between tumor ploidy and LOH load (*p* = 1.829 × 10^−7^). TMB showed an inverse association with both ploidy and the presence/absence of LOH, which became statistically significant after removing an outlier sample, #50 (*p* = 0.022 and *p* = 0.025, respectively). In the subset of patients (N = 19) treated with ICI agents, samples from R showed higher TMB (median = 21.91, IQR = 10.56–34.87 vs. median = 15.22, IQR = 4.22–37.13) and lower LOH load (median = 0, IQR = 0–1.257 × 10^14^ vs. median = 2.286 × 10^14^, IQR = 1.715 × 10^13^, 5.903 × 10^14^), and were more frequently diploid (N = 8 vs. N = 2) compared to samples from NR, although these associations were not statistically significant (all *p* > 0.05).

### 2.4. Genetic Layout Associated with Intrinsic and Acquired Resistance

Among our cohort of 36 patients, we analyzed 12 melanoma patients (4 *BRAF* V600+ and 8 *BRAF* V600−) for which matched pre-therapy and post-therapy biopsies were available, to identify potential genetic predictors of intrinsic and/or acquired resistance (Supplementary Figure S4). 

Overall, we found 20 melanoma driver gene variants shared between pre-therapy and matched post-therapy tumor samples (Table 1). 

Among those, 13 were reported as pathogenic in the COSMIC v96 database and 2 novel variants were considered potentially pathogenic because of their predicted impact on protein function (Table 1). Among the two novel potentially pathogenic mutations, only one was identified in the tumor samples of a NR patient (#63): the *FBXW7* p.Lys652 * nonsense mutation. Among the 13 mutations reported as pathogenic, which occurred in *BRAF*, *KIT*, *NRAS*, *HRAS*, *GNAQ*, *NF1*, *PPP6C*, *CTNNB1*, *ARID2*, and *IDH1*, two (p.Pro29Ser and p.Thr96Ser in the *RAC1* and *GNAQ* gene, respectively) were found in the same *BRAF* V600+ NR patient (#1). The same two genes (*RAC1* and *GNAQ*) were also both found mutated in another NR patient (#18). Interestingly, melanoma from this latter patient revealed a well-known *KIT* mutation (p.Leu576Pro), sensitive to Imatinib. Finally, a NR patient (#62) had the pathogenic p.Gln1313* mutation in the *ARID2* gene. Interestingly, AF of all mutations found in common between the two matched lesions (except p.Leu576Pro in *KIT* gene (#18), p.Val600Glu and p.Gln1313* in *BRAF* and *ARID2* genes (#62)) increased over time. 

Conversely, in 6 patients we identified 17 variants only in the second lesions (in *PREX2*, *KIT*, *EZH2*, *CNOT9*, *TP53*, *ARID2*, *GNAQ*, *NF1*, *RB1*, and *MTOR* genes). However, we checked by IGV the presence of these variants in the pre-therapy lesion, finding only nine variants acquired, of which only five were pathogenic COSMIC mutations. Notably, the remaining eight variants had been discarded by variant calling quality filters due to low allele frequency and poor coverage. Among those, five pathogenic mutations in the *KIT*, *EZH2*, *GNAQ*, and *RB1* genes were found in four patients, three of whom were NR (Table 2).

CNV analysis showed 23 CNVs in driver genes in common between the two matched melanoma lesions (Supplementary Table S5; Supplementary Figure S5) and 49 CNVs acquired in the second lesion only (Supplementary Table S6; Supplementary Figure S5). Interestingly, among the four *BRAF* V600+ patients, two (#1 and #39) showed acquired *BRAF* amplification, and one (#39) also showed a *PTEN* deletion, a known intrinsic resistance mechanism to TT (*BRAF*+*MEKi*), in common between the two matched lesions. However, *BRAF* amplification was confirmed by MLPA analysis in both lesions of the same patient (#1 and #39), supporting this finding as an intrinsic resistance mechanism. In both melanoma lesions from patient #63, harboring the potentially pathogenic *FBXW7* mutation (p.Lys652*), the second allele showed whole gene deletion, resulting in LOH and, thus, supporting the pathogenic role of this variant. Among the CNVs acquired in the second lesion, *RAC1* amplification was reported in the tumor sample of patient #1, who already harboring a pathogenic mutation (p.Pro29Ser) in the same gene.

### 2.5. Genomic Landscape of DNA Damage Repair (DDR) Deficiency Layout Associated with Intrinsic and Acquired Resistance

We determined the prevalence of DDR alterations across our cohort considering only exonic somatic Loss Of Function (LOF) variants with an AF of at least 10% in the tumor samples (Supplementary Table S7), identifying 66 LOF variants, 41 of which were unique. Interestingly, 66.7% (6/9) of *BRAF* V600− R patients had a tumor sample with at least 1 LOF variant vs. 37.5% (3/8) of *BRAF* V600− NR patients. An opposite trend was observed in *BRAF* V600+ patients. In the matched tumor samples, we found 50 DDR LOH in common between pre-therapy and post-therapy melanoma lesions (Supplementary Table S8). Interestingly, among the 12 matched tumor samples, only those from 2 patients (#39 and #63) revealed at least 1 LOH in common. Conversely, 309 LOH variants were only present in the post-therapy lesion (Supplementary Table S9).

### 2.6. Circulating Free DNA Mutation Profiles and Dynamic Changes during Treatment

**Figure 1:**
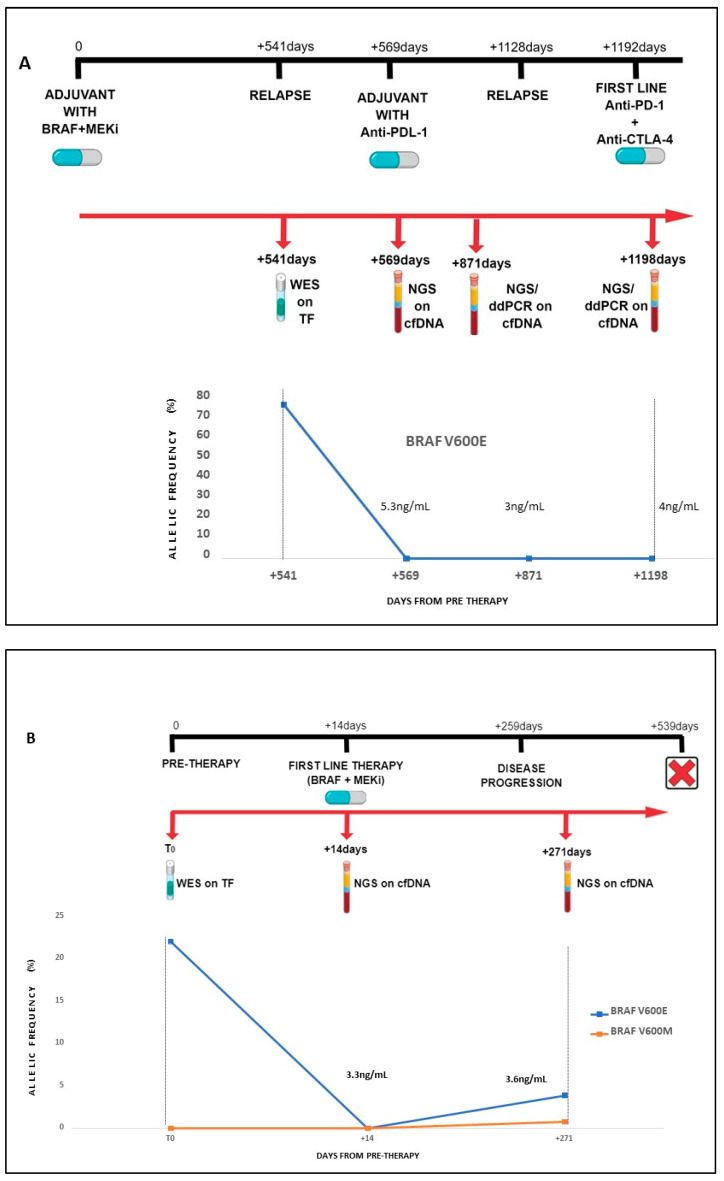

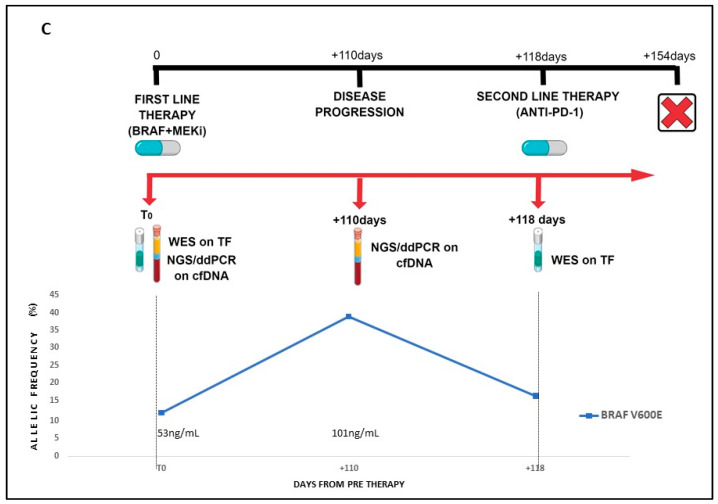
*BRAF* V600 mutation dynamic changes in longitudinal samples from non-responding patients. The figure shows *BRAF* V600 levels in circulating tumor DNA assessed longitudinally in three non-responding patients (#60 (**A**), #8 (**B**), and #62 (**C**)) by next-generation sequencing and/or droplet digital PCR compared with whole-exome sequencing data in fresh tumor tissue. Abbreviations: AF: Allele Frequency; WES: Whole-Exome Sequencing; ddPCR: droplet digital PCR; cfDNA: circulating free DNA; TF: Fresh Tissue.

cfDNA was extracted from 14 patients and sequenced by targeted NGS. For three of them, we were able to assess dynamic changes during treatment by analyzing circulating tumor DNA (ctDNA) at three (#60; Figure 1A) or two (#8 and #62; Figure 1B,C) consecutive time points through disease progression. The dynamic *BRAF* V600 profiles during treatment in the three patients with *BRAF* V600+ mutant melanoma (#60, #8, and #62) are shown in Figure 1A–C.

In case #60, a carrier of intraencephalic disease, *BRAF* V600 levels remained undetectable during progression and during therapy. In addition, total cfDNA levels remained low and constant over time (ranging from 3 to 5 ng/mL) (Figure 1A). In case #8, *BRAF* mutation was undetectable at the time of first-line therapy with *BRAF*+*MEKi*. Conversely, at the time of disease progression, *BRAF* p.Val600Glu was found in ctDNA, together with an additional mutation affecting the same codon (p.Val600Met), which could explain the progression to TT, although cfDNA level was low due to intraencephalic disease (Figure 1B). Finally, *BRAF* p.Val600Glu AF in ctDNA from patient #62 increased during first-line therapy with *BRAF*+*MEKi* (from 12% pre-treatment to 39% at the disease progression), as well as the total cfDNA level (from 53 ng/mL to 101.3 ng/mL) (Figure 1C).

The genomic features of variants, CNVs, and fusions included in the NGS panel were evaluated for each of the 14 patients (Supplementary Table S10). Interestingly, the median amount of total cfDNA was 13.5 ng/mL vs. 45.1 ng/mL in responder vs. non-responder patients.

**Figure 2:**
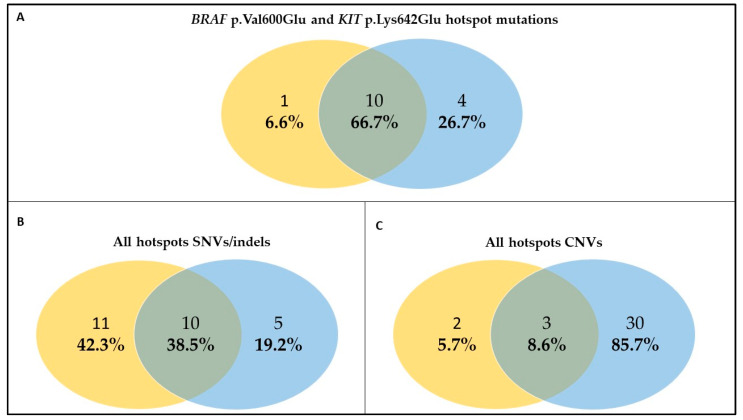
Concordance analysis between circulating free DNA and tumor tissue. The figure shows the concordance between cfDNA (yellow circle) and tumor tissue (blue circle) when considering only *BRAF* p.Val600Glu and *KIT* p.Lys642Glu hotspot mutations (**A**), all hotspot SNVs/indels (**B**), all hotspot CNVs (**C**). Abbreviations: SNVs: Single Nucleotide Variants; indels: insertions/deletions; CNVs: Copy Number Variations.

The hotspot mutations (*BRAF* p.Val600Glu and *KIT* p.Lys642Glu) detected by WES in tissue biopsies were also revealed on cfDNA by NGS panel in 66.7% of samples (Figure 2A). In four samples (#26, #3, #8_T1, and #60), there was a discordance between cfDNA and tissue biopsy. cfDNA sample #8_T1 did not reveal the *BRAF* p.Val600Glu present in the corresponding tissue, likely because this sample’s %LOD ranged from 1% to 1.2% due to low molecular coverage 443x). *BRAF* p.Val600Glu was absent in patient #26’s cfDNA, possibly due to a good response to *BRAF*+*MEKi* therapy observed in this patient. Conversely, the lack of the same mutation in cfDNA from patient #3 may be ascribed to the presence of not-tumor cfDNA confirmed by the presence of the p.Pro61Ala in the *SMO* gene (at an AF of 50%), as evidenced by the WES on PBMC of this patient. Finally, #60 cfDNA did not reveal the *BRAF* p.Val600Glu, but this patient was a stage IIIB NED treated for one and a half years with *BRAF*+*MEKi* adjuvant with a local relapse. 

Extending the analysis to all hotspot SNVs/indels and CNVs included in the NGS panel resulted in a drop in the concordance between cfDNA and tissue biopsy to 38.5% and 8.6%, respectively (Figure 2B,C). As expected, cfDNA analysis detected additional SNVs/indels in 42.3% of the samples. Compared to *BRAF* and *KIT* hotspot analysis, discordant SNVs/indels in other genes were predominantly found in cfDNA vs. tissues (66.7% vs. 26.7% and 38.5% vs. 19.2%, respectively). Conversely, the proportion of CNVs was higher in cfDNA vs. tissue (85.7% vs. 5.7%).

Interestingly, CDK4, CDK6, and EGFR amplifications were shared by both cfDNA and tissue biopsy in all the samples, while MET amplification was found only in two cfDNA samples (Supplementary Table S10 and Figure 2C).

### 2.7. Characterization of Germline Pathogenic Variants (PVs) by Whole-Exome Sequencing (WES)

The analysis of 166 cancer predisposition genes in germline DNA identified 83 exonic non-synonymous variants in 29 out of 36 patients (Supplementary Data). Three pathogenic variants (PVs) in melanoma predisposition genes were found (*MITF* p.Glu318Lys and *CDKN2A* p.Gly101Trp in #56 and *MITF* p.Glu318Lys in #62). In addition, patient #63 carried the p. Ser1993ArgfsTer23 PV in the *ATM* gene, which has been recently associated with melanoma susceptibility [24]. Overall, three patients carried four melanoma predisposition PVs, resulting in a germline PV frequency of 8.3%. All four variants were confirmed by Sanger sequencing.

## 3. Discussion

This study aimed to assess the genetic layout of 36 consecutive melanoma patients (stage III/IV) treated with TT or ICI agents in a real-world setting, classified as Responders (R) or Non-Responders (NR), and followed-up during therapy. Mutations and CNVs in melanoma driver genes, mutations and LOH in DDR genes, total TMB, and LOH load were analyzed. According to the four main melanoma genetic subtypes established by The Cancer Genome Atlas (TCGA), we found 52.8% (19/36) *BRAF*-mutant, 30.6% (11/36) *NRAS*-mutant, 8.3% *NF1*-mutant, and 13.9% (5/36) triple wild-type melanomas [25]. Among the *BRAF*-mutant subset, we found one sample with *NRAS* Q61 mutation and another sample with an *NF1* mutation. Another sample showed coexisting *NRAS* and *NF1* mutations, supporting the three-group melanoma classification (*BRAF*-mutant, *RAS*-mutant, non-*BRAF*-mutant/non-*NRAS*-mutant) [26]. After *BRAF*, *NRAS*, and *NF1*, the most frequently mutated driver genes were *TP53* (27.8%), *ARID2* (19.4%), *KIT* (16.7%), *PREX2* (13.9%), *RAC1* (13.9%), and *FBXW7* (11.1%) (Supplementary Figure S1). *TP53* is the most frequently mutated gene in human cancer, with a frequency of 36.8% in the TCGA database and a significant prevalence of missense mutations. The *TP53* mutation rate in our study is in line with literature data [27]. In the *BRAF* V600+ subset (N = 19), melanoma driver gene mutations and CNVs were higher in tumors from NR than those from R, suggesting a genetic mechanism promoting tumor escape from TT, although the difference was not statistically significant. In contrast, in the *BRAF* V600− subset (N = 19), no differences were found for driver melanoma gene mutations, CNVs, and interferon pathway gene mutations. However, CNV analysis of the *HLA-A*, *HLA-B*, *HLA-C*, *TAP2*, *PD-1* (PDCD1), *PDL1* (CD274), and *PDL2* (PDCD1LG2) genes, as possible determinants of response in this cohort, led to the identification of six tumors (four from R patients, 66%) carrying at least one amplification in one of these genes, confirming literature data [28,29,30]. Our WES data revealed no difference in TMB between R and NR patients, while *BRAF* V600− responders showed a doubled TMB compared to NR, in keeping with other studies [31]. In our cohort, we observed tumor aneuploidy in the majority of metastases, a common finding in advanced human cancers [32], which significantly correlated with extensive LOH. In addition, both ploidy and LOH load showed an inverse association with TMB, which became statistically significant after removing an outlier sample (#50). One potential role of LOH, caused by extended tumor aneuploidy or whole genome doubling, could be to determine tumor biological advantage by eliminating multiple deleterious mutations and reducing immunogenicity through the deletion of neoantigens [33]. In the subset of patients treated with ICI agents, samples from R showed higher TMB and lower LOH load, and were more frequently diploid, compared to samples from NR, although these associations were not statistically significant. Moreover, WES data were obtained from matched pre-therapy and post-therapy biopsies in 12 melanoma patients, focusing on acquired and intrinsic resistance mechanisms, as well as genetic determinants of response. Mutational profiles revealed commonly known and novel potential intrinsic/acquired resistance driver gene variants. Among these, *RAC1*, *FBXW7*, *GNAQ* mutations, and *BRAF*/*PTEN* amplification/deletions, were found in 42% and 67% of patients, respectively. Moreover, we found a novel potentially pathogenetic variant in the *FBXW7* gene (p.Lys652*) shared by both pre-therapy and post-therapy matched biopsies (Table 2). *FBXW7* is a critical tumor suppressor gene and a member of the F-box protein family, ubiquitin ligase complex, that controls proteasome-mediated degradation of oncoproteins such as cyclin E, c-Myc, Mcl-1, mTOR, Jun, Notch, AURKA, and STAT2 [34,35,36]. *FBXW7* LOF in several human cancers has multiple clinical implications, including prognostic value; for instance, rapamycin has been proven to inhibit *FBXW7*-deficient breast cancer cells by mTOR inhibition [35,37]. Moreover, in another study, *FBXW7α* deficiency leads to HSF1 (Heat shock factor 1) accumulation and subsequent activation of the invasion-supportive transcriptional program and metastatic potential of human melanoma cells [38]. It may be hypothesized that the novel mutation found in our study might have conferred resistance to immunotherapy in this patient, belonging to the NR *BRAF* V600− subset [39]. *FBXW7* loss has been recently described to confer radiosensitivity to cancer cells through a mechanism that leads to the accumulation of TP53 [40]. Literature data have already reported *FBXW7* as a gene correlated with acquired resistance to therapy [41,42]. Overall, we think that this LOF mutation gene (undergoing LOH) was associated with intrinsic resistance in this patient. To the best of our knowledge, this is the first identification of an *FBXW7* LOF mutation associated with intrinsic resistance. Two other mutations (p.Pro29Ser in the *RAC1* gene and p.Thr96Ser in the *GNAQ* gene), reported as pathogenic in the COSMIC database, were concomitantly found in one *BRAF* V600+ NR patient (#1). This patient had no clinical benefit from first-line TT, and, after a massive disease progression, he started a II-line therapy anti-*PD-1* which, equally, provided no benefit (progression free survival (PFS) of 0.97 months). Death occurred one month after the start of anti-*PD-1*. The paradoxical activation of the MAPK/ERK pathway through p.Pro29Ser mutation in the *RAC1* gene is a recognized mechanism responsible for primary/acquired resistance in melanoma [43]. However, the mechanisms by which this gene confers resistance have not been clearly defined. Although *RAC1* inhibitor drugs are not currently available in clinical practice, SRF/MRTF inhibitors in combination with *BRAF* inhibitors have recently been shown to be effective in the treatment of *BRAF* mutant melanoma cells with a co-occurring *RAC1* P29S mutation [43]. However, in melanoma, targeting *RAC1* is not currently being tested among available clinical trials (https://clinicaltrial.gov, accessed on 20 February 2023). The *GNAQ* p.Thr96Ser mutation has been already reported in patients with Natural killer/T cell lymphoma (NKTCL). This mutation causes loss of Gnaq protein function leading to, as demonstrated by increased binding to G beta-gamma protein in cell culture, high Erk and Akt phosphorylation in cultured cells and xenograft tumors, and increased tumor growth in mouse models compared to the wild-type *GNAQ* [44,45]. *RAC1* and *GNAQ* were also found concomitantly mutated in another NR patient (#18) with a known *KIT* mutation (p.Leu576Pro) sensitive to Imatinib. Indeed, patient #18 started I-line therapy with an anti-*PD-1* agent. Disease progression occurred 6 months later, after which the patient underwent II-line chemotherapy. Imatinib was not considered as a possible therapeutic option because *KIT* mutational status was not assessed at the time of the therapy selection. Finally, the TT NR patient (#62) had the *ARID2* p.Gln1313* pathogenic mutation already described in melanoma [46]. Cancers with inactivating *ARID2* mutations are more sensitive to *PD-1* blockade, as well as to other types of immunotherapies [47]. Moreover, higher sensitivity to different DNA-damaging therapies has been observed ARID2-deficient non-small cell lung cancer cells, likely due to ARID2 involvement in DNA repair [48]. *PTEN* deletion is one of the best-known molecular mechanisms of intrinsic resistance to *BRAF* inhibitors, and was present in 42.1% of our *BRAF* V600+ subset, namely, in tumors from two R (#2 and #39) and five NR (#3, #14, #19, #56, #10) patients [49]. In melanoma, the reactivation of the MAPK pathway during *BRAF*/MEK blockade can occur through several mechanisms, including amplification of the *BRAF* gene. *BRAF* amplification was indeed present in tumors from 3/19 patients (15.8%) belonging to the *BRAF* V600+ subset. This is in line with previously reported WES data which showed acquisition of resistance to *BRAF* inhibitors due to *BRAF* gene amplification in around 20% of melanoma patients [50]. Only one *BRAF* V600+ patient (#39), whose melanoma harbored both *BRAF* amplification and *PTEN* deletion, responded to *BRAF*+*MEKi*. This unexpected result suggests that this patient’s response was due to unknown additional mechanisms, such as gene fusions or transcriptional events. 

Germline susceptibility variants may have therapeutic implications. In our study cohort, we found three PVs in melanoma predisposition genes in two patients (*MITF* p.Glu318Lys and *CDKN2A* p.Gly101Trp in #56 and *MITF* p.Glu318Lys in #62). Inherited PVs in the *CDKN2A* tumor suppressor gene are among the strongest risk factors for cutaneous melanoma [51]. Recent studies demonstrated that response to *BRAF*+*MEKi* in patients with germline *CDKN2A* PVs was not inferior to data from clinical trials and real-world studies [52], while the response rate to immunotherapy was superior in *CDKN2A* PVs carriers, likely due to an increased tumor mutational load [53]. The patient with *BRAF* (#56) V600+ mutant melanoma who carried both *CDKN2A* and *MITF* germline PVs did not respond to adjuvant therapy with TT nor to first-line treatment with ICI. The same germline *MITF* PV was found in another patient (#62) with *BRAF* V600+ melanoma non-responding to both first-line and second-line therapies with TT and ICI agents, respectively. This finding prompts further investigations into the response to therapy in melanoma predisposition genes’ germline PVs carriers. Finally, we identified a novel PV in the *ATM* gene (p.Ser1993ArgfsTer23) in a *BRAF* V600− patient (#63) [54]. Following the identification of *ATM* germline variants in melanoma patients [55] and the emergence of *ATM* as a melanoma Genome-Wide Association Study hit [56], a multicentric international study on 2105 melanoma cases proposed *ATM* as an intermediate-risk melanoma predisposition gene [24]. In addition, a recent study reported loss of ATM expression in melanoma samples from germline *ATM* PV carriers, supporting the role of this gene in melanoma predisposition [54]. Despite being unselected for melanoma family history, our cohort revealed a high germline PV rate (8.3%). None of the included patients reported either personal history of multiple primary melanoma or family history of melanoma and/or associated cancers. Interestingly, this figure is comparable to the 9.5% germline PV rate that we recently observed in a high-risk melanoma cohort enrolled in a 5-year time span and tested by a multigene panel within the Italian Melanoma Intergroup [57], supporting the relevance of the germline testing secondary to somatic WES. Moreover, we evaluated the potential of cfDNA analysis as a non-invasive surrogate for tissue biopsy for the identification of hotspot mutations (*BRAF* p.Val600Glu and *KIT* p.Lys642Glu mutations), and found good concordance between liquid and solid biopsies (Figure 2A). We can formulate two main hypotheses to explain the lack of concordance between cfDNA and DNA extracted from tissue biopsy. First, the presence of a low disease burden, as observed in patient #60, could result in low levels or even absence of circulating DNA; alternatively, a response to TT could result to the absence of *BRAF* mutant DNA, which might be the case for patient #26, for instance. Second, the presence of exclusively encephalic metastases (#3, #8, and #60), effectively isolated by an intact blood brain barrier, could explain low levels of ctDNA in peripheral blood [58]. Both these scenarios necessarily raise questions about the limits of ctDNA in terms of the lower sensitivity limit of the method and reliability depending on tumor burden and metastases sites. As ctDNA levels are thought to reflect tumor burden, a decrease in ctDNA during therapy may be a sign of treatment efficacy. We assessed whether longitudinal changes in ctDNA of three patients could supplement or improve RECIST-based measures for clinical decision making. Indeed, for patients #62 and #8, ctDNA analysis during treatment was predictive of disease progression or an additional *BRAF* mutational event, even in the case of intra-encephalic disease. As expected, concordance between liquid and solid biopsies decreased when all hotspot SNVs/indels and CNVs were included in the assessment (Figure 2B,C). Compared to *BRAF* and *KIT* hotspot analysis, discordant SNV/indels were predominantly found in cfDNA vs. tissues (Figure 2B). Conversely, the opposite trend was observed for CNV analysis in line with recent data [59]. This study has several limitations. The main limitation is represented by the low number of thoroughly analyzed patients and the heterogeneity of treatments. Unfortunately, collecting consecutive biopsies from patients before systemic therapy and at each disease can be arduous depending on the clinical context, the (sometimes inaccessible) sites of progression (i.e., brain), and the patient’s will. On the other hand, this case series is entirely real-world, reflecting current therapeutic opportunities and the possibility and extent of molecular tumor characterization in terms of biopsy accessibility and availability of material. This study is preliminary to the analysis of the intricate interplay between tumor cells, tumor microenvironment, and the immune system, including local and systemic factors, which are likely to modulate the efficacy of different therapies. All these factors are complex and dynamic, and multi-layer integrated studies of real-world melanoma cohorts longitudinally followed up during medical therapy are an opportunity to yield major insights into this interplay.

## 4. Materials and Methods

### 4.1. Melanoma Patient’s Cohort

Based on the availability of a fresh tissue biopsy, 36 melanoma patients (19 *BRAF* V600+ and 17 *BRAF* V600− patients; stage III/IV) were consecutively and prospectively recruited (at first access or at relapse) at the IRCCS Ospedale Policlinico San Martino and treated in the adjuvant or advanced disease setting according to clinical practice (with ICI agents (*PD-1* and/or CTLA-4 inhibitors) and/or TT (*BRAF*+*MEKi* or *KIT* inhibitors) [60]. Tumor reassessments were performed according to Response Evaluation Criteria in Solid Tumors (RECIST) 1.1 and immune-RECIST (iRECIST) criteria [61]. The clinical benefit was assessed for each patient according to the type of treatment and setting, classifying patients as Responders (R) and Non-Responders (NR).

The clinical benefit from adjuvant treatment was defined as the absence of disease recurrence at the follow-up cut-off. In patients who received first-line treatment for advanced disease with *BRAF*+*MEKi*, clinical benefit was defined by Progression Free Survival (PFS) > 11 months, according to COMBI-d study results [11]. In patients treated with *PD-1* inhibitors (monotherapy or in combination with CTLA-4 or *BRAF*+*MEKi*) clinical benefit was defined by a Best Overall Response (BOR) of Stable Disease (SD), Partial Response (PR), or Complete Response (CR). 

In selected cases, treatment was continued beyond disease progression. The clinical characteristics of the patients are reported in Supplementary Table S11.

For each patient included in the study, the somatic DNA of the pre and/or post therapy metastasis was extracted from fresh tissue. In the absence of fresh tissue biopsy from the pre-therapy melanoma, DNA was extracted from six archival FFPE sections. In addition, peripheral blood was taken for germline DNA extraction from all patients. 

Tumor tissue samples were selected and revised based on tissue quality and tumor cellularity by the pathology team. The study was approved by the local IRB (046REG2017), and written informed consent was obtained from all the patients.

### 4.2. DNA and Circulating Free DNA (cfDNA) Extraction

Genomic DNA (gDNA) was extracted from peripheral blood using the Diatech MagCore^®^ HF16Plus (RBC Bioscience, New Taipei City, Taiwan) with the Genomic DNA Large Volume Whole Blood kit. gDNA purity was assessed with a Nanodrop 2000 spectrophotometer (Thermo Fisher Scientific, Carlsbad, CA, USA) to measure the whole absorption spectrum (220–750 nm) and calculate absorbance ratios at 260/280 and 260/230. gDNA yield was evaluated by fluorometric quantitation using Qubit^®^ Fluorometer (Life Technologies Corporation, San Francisco, CA, USA).

Somatic DNA from FFPE was extracted from the tumor sections using the Genomic DNA FFPE One-Step Kit for Diatech MagCore^®^ HF16Plus extractor (RBC Bioscience) according to the manufacturer’s instructions. 

Somatic DNA from fresh tissue biopsy was isolated using a DNeasy^®^ Blood & Tissue Kit (QIAGEN, Valencia, CA, USA). Quantity and purity of the tumor gDNA were examined by SPECTROstar Nano (BMG Labtech, Offenburg, Germany) to measure the whole absorption spectrum (220–750 nm) and calculate absorbance ratios at both 260/280 and 260/230. Moreover, all somatic and germline samples were quantified by Qubit^®^ 2.0 Fluorometer (Invitrogen, Carlsbad, CA, USA) and Agilent 2200 TapeStation system using the Genomic DNA ScreenTape assay (Agilent Technologies, Santa Clara, CA, USA). 

cfDNA was isolated from 1–5 mL of plasma using MagMAX™ Cell-Free DNA Isolation Kit according to the manufacturer’s instructions (Thermo Fisher Scientific) and quantified using the Qubit^®^ dsDNA HS Assay Kit on the Qubit 2.0 fluorometer (Thermo Fisher Scientific). The purity and quantity of DNA size fragments were analyzed by the Agilent High Sensitivity DNA Analysis Kit (Agilent Technologies) using a TapeStation 2200 instrument (Agilent Technologies).

### 4.3. Whole-Exome Sequencing (WES)

gDNA from peripheral blood and somatic DNA from fresh tumor tissue and/or FFPE were subjected to WES at a coverage of 100× and 300×, respectively.

Nextera Flex for Enrichment solution (Illumina, San Diego, CA, USA) combined with ‘SureSelect Human All Exon V7’ probes (Agilent Technologies) was used for library preparation and exome enrichment, targeting 50 Mb of human exonic content. All samples were quantified and quality tested using the Qubit 2.0 Fluorometer (Invitrogen, Carlsbad, CA, USA) and Agilent 2100 Bioanalyzer (Agilent Technologies). Libraries were sequenced on NovaSeq 6000 (Illumina, San Diego, CA, USA) in 150 pair-end mode. Raw data were first processed for both format conversion and de-multiplexing by the Bcl2Fastq 2.0.2 version of the Illumina pipeline (https://support.illumina.com/content/dam/illumina-support/documents/documentation/software_documentation/bcl2fastq/bcl2fastq2-v2-20-software-guide-15051736-03.pdf, accessed on 25 January 2023). Adapter sequences were masked with Cutadapt v1.11 from raw fastq data using the following parameters: anywhere (on both adapter sequences)—overlap 5—times 2—minimum-length 35—mask-adapter [62]. Subsequently, Illumina DRAGEN Germline 3.5.7 and Somatic Pipelines 3.5.7 were used to map reads to GRCh38/hg38 assembly and identify germline and somatic tumor/normal matched pair variants, respectively (https://emea.illumina.com/products/by-type/informatics-products/basespace-sequence-hub/apps/dragen-germline.html; https://emea.illumina.com/products/by-type/informatics-products/basespace-sequence-hub/apps/edico-genome-inc-dragen-somatic-pipeline.html, accessed on 25 January 2023) [63,64]. Variants were functionally annotated by Annovar [65]. Single nucleotide polymorphisms (SNPs) and small insertions and deletions (indels) summary reports contain variant coordinates, base pair changes, amino acid change annotation, and functional annotation, including the clinical significance of a sequence variation to human health, population frequencies, and a series of scores (SIFT, PolyPhen, LRT, MutationTaster, etc.) as well as Human Phenotype Ontology (HPO) and other information helpful for variant prioritization. 

TMB of each tumor sample was calculated using the total number of PASSING filter non-synonymous somatic mutations (SNPs and indels) divided per mega-base of callable somatic regions included in the total genomic target region captured with the exome assay (35 Mb).

CNVkit 0.9.7 was used to detect somatic CNVs [66]. BAM files of the 36 germline melanoma patients were used to generate a reference of per-bin read count. Similarly, tumor samples were bin counted using default parameters, and each was compared to the reference normalized 0-centered signal. For each tumor sample, bins were segmented using default parameters (circular binary segmentation). Bins with log2 normalized coverage values below −15 were removed. CNAs call thresholds on log2 parameters were as follows: <−1.1 = 0, <−0.4 = 1, <0.4 = 2, <0.8 = 3. Calls with log2 confidence intervals overlapping zero were removed. LOH was performed using ASCAT [67]. Bona fide LOH events were defined as a region with a number of copies of the minor allele equal to zero. LOH load was calculated as the sum of bona fide LOH events (genomic regions with a number of minor alleles equal to zero) divided by the total exonic regions, per megabase. The melanoma driver, the interferon-gamma pathway, and DDR gene mutation analysis were performed considering exons somatic variants with ‘PASSING’ filters (missense, indel, stop mutations) (Supplementary Figure S1) in the R vs. NR.

Melanoma driver genes [22], interferon-gamma pathway [68], DDR genes [69] selected for mutation, CNV and LOH analysis, and the 166 cancer predisposition genes for the germline analysis [70] are reported in Supplementary Figure S6.

The tumor Single-Base Substitution (SBS) signatures were calculated starting from the Variant Call Format (VCF) file of the somatic tissue samples (subtracted from the germline variants resulting from the germline analysis of the corresponding patient) using three different tools: DeconstructSigs, Signature Multivariate Analysis (SigMa), and SigProfiler v3.2 [71,72,73]. 

### 4.4. Next-Generation Sequencing (NGS) and Droplet Digital PCR (ddPCR) Analysis on Circulating Free-DNA

Targeted libraries were amplified using Oncomine™ Pan-Cancer Cell-Free Assay (Thermo Fisher Scientific), which detects hotspot mutations, small indels, copy number changes, and gene fusions across 52 genes. In particular, this assay includes 177 amplicons covering 980 key hotspot mutations in 44 known cancer genes (*AKT1*, *ALK*, *APC*, *AR*, *ARAF*, *BRAF*, *CHEK2*, *CTNNB1*, *DDR2*, *EGFR*, *ERBB2*, *ERBB3*, *ESR1*, *FBXW7*, *FGFR1*, *FGFR2*, *FGFR3*, *FGFR4*, *FLT3*, *GNA11*, *GNAQ*, *GNAS*, *HRAS*, *IDH1*, *IDH2*, *KIT*, *KRAS*, *MAP2K1*, *MAP2K2*, *MET*, *MTOR*, *NRAS*, *NTRK1*, *NTRK3*, *PDGFRA*, *PIK3CA*, *PTEN*, *RAF1*, *RET*, *ROS1*, *SF3B1*, *SMAD4*, *SMO*, and *TP53*) and CNAs in 12 genes (*CCND1*, *CCND2*, *CCND3*, *CDK4*, *CDK6*, *EGFR*, *ERBB2*, *FGFR1*, *FGFR2*, *FGFR3*, *MET*, and *MYC*). Furthermore, it allows the identification of de novo variants in the TP53 gene with frequency > 1%.

The recommended cfDNA input amount for the Oncomine assay is 20 ng. However, as low as 2 ng of cfDNA may be sufficient to evaluate circulating tumor DNA (ctDNA) with this assay (Thermo Fisher Scientific). Patients cfDNAs (range 2–20 ng per reaction) were employed to prepare manually targeted libraries following manufacturer’s instructions, quantified with the High Sensitivity DNA Analysis Kit (Agilent Technologies) using a TapeStation 2200 instrument (Agilent Technologies), diluted to 100 pM, and pooled for automated templating with an Ion 540™ kit for the IonChef Instrument. Sequencing was performed with the GeneStudio S5 system and Ion 540™ chips (4 samples/chip).

Sequence data were processed using the Torrent Suite 5.10.1 pipeline software optimized for the Ion Torrent platform to perform raw data analysis and base calling, remove low-quality reads, and make alignments to the human genome (GRCh37/hg19). Variant calling was performed with Ion Reporter Server 5.12 and the software Oncomine™ TagSeq S540 Liquid Biopsy—w2.4—Single Sample detecting and annotating low-frequency variants, including SNPs/InDels (down to 0.1% limit of detection), fusions, and copy number variations (CNVs). The hotspot calls were reviewed by uploading each VCF file on (IGV) (http://www.broadinstitute.org/igv, accessed on 25 January 2023) [74]. An average of 82 million total reads was generated and mapped to the reference genome per library, and 95% of the mapped reads were on a target. The mean depth of coverage ranged from 23,165× to 96,908× (average of 59,021×). The uniformity of each library, which is the percentage of amplicons (bases) covering more than 20× of the mean amplicon (base) coverage, ranged from 98.1% to 99.6%. Only one cfDNA sample (#8_T1), for which a lower cfDNA input was used (2 ng), had a molecular coverage lower than 2000×. 

The *BRAF* V600 status was also evaluated by ddPCR for two patients on cfDNA (#62_t1, #62_t2, #60_t2, and #60_t3). The presence of the *BRAF* V600 mutation and its allele frequency in the ctDNA was evaluated by the QX200 droplet digital PCR™ (ddPCR) system (Bio-Rad Laboratories, Inc., Hercules, CA, USA) using the “ddPCR *BRAF* V600 Screening Kit” (BioRad), able to detect p.Val600Glu, p.Val600Lys, and p.Val600Arg mutations in a single run. 

### 4.5. BRAF Multiplex Ligation-Dependent Probe Amplification (MLPA) Analysis

All *BRAF*-mutated samples included in this study that revealed a CNV in the *BRAF* gene by WES were validated by Multiplex Ligation-dependent Probe Amplification (MLPA) analysis using the SALSA MLPA Probemix P298 *BRAF*-HRAS-KRAS-*NRAS* (MRC Holland BV, Amsterdam, the Netherlands). This probemix contains 57 probes for the detection of deletions and/or duplications in the *RAS* genes (*HRAS*, *KRAS*, and *NRAS*) and the *BRAF* gene, and includes one probe specific for the *BRAF* p.Val600Glu (c.1799T>A) mutation and two probes for *KRAS* c.34G and c.35G, both located in codon 12.

The MLPA assay was performed according to the manufacturer’s instructions (MRC Holland BV). The MLPA products were separated by capillary electrophoresis in an automated sequencer (ABI 3130XL Genetic Analyzer, Applied Biosystems). The results were interpreted using the Coffalyser.Net software (MRC Holland BV). Ratios < 0.75, 0.75–1.30, and >1.3 were considered to indicate deletion, normal, and duplication, respectively.

### 4.6. TERT Core Promoter Mutational Status

Mutational status of the *TERT* core promoter was determined in the tumor samples by Polymerase Chain Reaction (PCR) and Sanger sequencing between genomic positions 1294925 and 1295198. In detail, we amplified the *TERT* promoter (located on chromosome 5) target region (LRG_343, NG_009265.1, NM_198253.3) using forward and reverse primers: TERT_Forward: gTC CTg CCC CTT CAC CTT and TERT_reverse: AgC ACC TCg Cgg TAg Tgg. The specific primer pairs were designed using the Primer3 algorithm (https://primer3plus.com, accessed on 25 January 2023) [75], a primer designing tool. The PCR reactions were performed by amplifying 40 ng of tumor gDNA in a final volume of 15.5 μL containing 200 mol/L dNTPs, 10× Taq buffer, 0.322 μM of each PCR primer, and 1.5 U of Taq Hot Start (Qiagen). The PCR program consists of 10 min at 95 °C and 35 cycles with 30 s at 95 °C, 30 s at 60 °C for annealing temperature, and 30 s at 72 °C, followed by 5 min at 72 °C. Purified products were sequenced using the same primers of the PCR amplification with the BigDye Terminator v1.1 cycle sequencing kit (Applied Biosystems) under the following conditions: 1 μL BigDye Terminator v1.1, 2 μL sequencing buffer 5×, 3.2 pmol forward or reverse primer, 1.5 μL PCR purified product, and 4 μL sterile water to a final reaction volume of 10.5 μL. Cycle sequencing was performed using an initial denaturation step at 96 °C for 10 s followed by 25 cycles at 96 °C for 10 s, and 60 °C for 3 min on GeneAmp^®^ PCR System 9700 (Applied Biosystems). The sequencing products were separated by capillary electrophoresis in an automated sequencer (ABI 3130XL Genetic Analyzer, Applied Biosystems) with a 36 cm length capillary and POP-7™ polymer, according to the manufacturer’s instructions. Data were analyzed with Sequencing Analysis Software version 5.3.1 (Applied Biosystems). The two most frequently identified variations within the *TERT* promoter gene region at genomic positions 1295228 and 1295250, known as C228T and C250T, respectively, were analyzed. These mutations are located at −124 and −146 bp upstream of the ATG start codon and were considered for analysis.

### 4.7. Statistical Analysis

To assess the median difference of a numerical variable (TMB, LOH load, number of DDR genes with LOH) between two groups, we used the Wilcoxon rank sum test for unpaired samples (Mann–Whitney’s U test). We used the Fisher exact test to analyze differences in the distribution of a categorical variable (COSMIC signatures, absence/presence of *TERT* promoter mutation, absence/presence of LOH, ploidy) between two groups, computing odds ratios and 95% confidence intervals in the case of 2 × 2 contingency tables. All tests were two sided, and a *p*-value cut-off of 0.05 was considered for statistical significance. All analyses were conducted within the R computational environment, using the following packages: readxl, tidyverse, patchwork, and ggmosaic [76,77,78,79].

## 5. Conclusions

This study, which integrated fresh tissue WES and plasma cfDNA analysis, aimed to comprehensively assess the genetic layout of metastatic melanoma in a consecutive real-world setting of patients undergoing treatment. Indeed, identifying mutations associated with primary resistance to TT and ICB agents plays a progressively increasing role in clinical practice. The early recognition of these patients would allow us to better define the most suitable therapeutic path, sparing them therapeutic toxicity without clinical benefit. In addition, a thorough characterization of the molecular profile could allow access to specific therapies that, in clinical practice, could be underused (as in a *KIT* previously undetected carrier). Resistance-associated mutational profiles showed known and novel potential melanoma drivers and resistance gene mutations. Indeed, in this study, at least one potential intrinsic/acquired driver resistance variant and/or amplification/deletion was found in almost all cases. In patients treated with ICB agents, samples from responders revealed higher TMB and lower LOH load with diploid tumor compared to non-responders. Finally, secondary germline findings support further investigations into the response to therapy in carriers of germline pathogenic variants in melanoma predisposition genes. Although our real-world study presents a small number of samples analyzed, which limited statistical analysis, ploidy and LOH were inversely associated with TMB. These findings pave the way to discriminating patients who could benefit from one treatment over another using larger cohorts of patients.

## Figures and Tables

**Table 1:** Melanoma driver gene variants detected by Whole-Exome Sequencing (WES) in common between pre-therapy and post-therapy melanoma lesions.

Patient ID	*BRAF* V600 Status	R	Gene	Ref seq	aa Change	Codon Change	AF %
#1	+	n	*RAC1* *	NM_018890.4	p.Pro29Ser	c.85C>T	4.2 to 13.8
			*GNAQ* *	NM_002072.5	p.Thr96Ser	c.286A>T	3.0 to 66.7
#62	+	n	*ARID2* *	NM_152641.4	p.Gln1313 *	c.3937C>T	27.1 to 7.0
#20	−	y	*NRAS* *	NM_002524.3	p.Gln61Arg	c.182A>G	6.5 to 42.0
			*HRAS*	NM_005343.4	p.Pro140Thr	c.418C>A	20.4 to 41.2
#21	−	y	*NRAS* *	NM_002524.3	p.Gln61Arg	c.182A>G	25.0 to 50.0
			*NF1* *	NM_001042492.3	p.Ser2093Phe	c.6278C>T	30.0 to 45.2
			*PPP6C* *	NM_001123355.1	p.Arg301Cys	c.901C>T	56.4 to 96.6
			*CTNNB1* *	NM_001098209.2	p.Ser45Pro	c.133T>C	27.8 to 49.3
#7	−	y	*IDH1* *	NM_005896.3	p.Arg132Cys	c.394C>T	37.5 to 23.0
			MAP2K2 **	NM_030662.3	p.Leu102_Ile107del	c.304_321delCTGATC CACCTTGAGATC	65.8 to 45.1
#63	−	n	*NRAS* *	NM_002524.3	p.Gln61Lys	c.181C>A	69.8 to 74.0
			FBXW7 **	NM_001349798.2	p.Lys652 *	c.1954A>T	64.2 to 71.4
#18	−	n	*KIT* *	NM_000222.2	p.Leu576Pro	c.1727T>C	88.8 to 86.4
			*TP53*	NM_000546.5	p.Pro27Ser	c.79C>T	48.8 to 52.2
			*RAC1* *	NM_018890.4	p.Pro29Ser	c.85C>T	13.1 to 36.0
			*GNAQ*	NM_002072.5	p.Gly64Arg	c.190G>A	10.3 to 15.5
#57	−	n	*BRAF* *	NM_001374258.1	p.Leu624Phe	c.1870C>T	29.2 to 60.8
			*BRAF* *	NM_001374258.1	p.Gly509Ala	c.1526G>C	32.8 to 58.7
			*KIT* *	NM_000222.2	p.Lys642Glu	c.1924A>G	20.7 to 47.9

Abbreviations: R: Response; no: no; y: yes; *: Pathogenic Mutations; **: Potentially pathogenic mutations; AF: Allele Frequency.

**Table 2:** Melanoma driver gene pathogenic mutations detected by Whole-Exome Sequencing (WES) acquired in the post-therapy melanoma lesions.

Patient ID	*BRAF* V600 Status	R	Gene	Ref seq	aa Change	Codon Change	AF %
#42	+	y	*KIT* *	NM_000222.2	p.Met541Leu	c.1621A>C	22.7
			*EZH2* *	NM_004456.4	p.Tyr646Asn	c.1936T>A	16.7
#1	+	n	*GNAQ* *	NM_002072.5	p.Tyr101 *	c.303C>A	66.7
#62	+	n	*KIT* *	NM_000222.2	p.Met541Leu	c.1621A>C	46.5
			*RB1* *	NM_000321.2	p.Asn123Asp	c.367A>G	49.1
#34	−	n	*GNAQ* *	NM_002072.5	p.Tyr101 *	c.303C>A	36.4
			*GNAQ* *	NM_002072.5	p.Thr96Ser	c.286A>T	33.3

Abbreviations: R: Response; n: no; y: yes; *: Pathogenic Mutations; AF: Allele Frequency.

## Data Availability

The data presented in this study are available upon request at Synapse (https://doi.org/10.7303/syn47354875, accessed on 25 January 2023).

## Supplementary Materials

The following supporting information can be downloaded at: https://www.mdpi.com/article/10.3390/ijms24054302/s1.
