# Supplementary material for: Whole-Exome Sequencing and cfDNA Analysis Uncover Genetic Determinants of Melanoma Therapy Response in a Real-World Setting
Source: Int J Mol Sci. 2023 Feb 21;24(5):4302. doi: 10.3390/ijms24054302 (PMC10002464; doi:10.3390/ijms24054302)
Supplement: Supplementary file 1 [file ijms-24-04302-s001.zip › Supplementary Tables_proof.pdf]

**Supplementary Table S1.** Real-world cohort of fresh tissue biopsies belonging to 36 melanoma patients.

| ***BRAF* V600+ Patients** | **Total N=19** | **R** | **NR** |
|---|---|---|---|
| SEX | F | 4 | 6* |
| | M | 4 | 5 |
| PRE-THERAPY BIOPSY | | 1 | 9* |
| POST-THERAPY BIOPSY | | 7 | 2 |
| TUMOUR ONSET MEDIAN | | 55.5y | 54.1y |
| THERAPY | BRAF+MEKi | 6 | 7 |
| | Anti-PD-1 | 2 | 1 |
| | Adjuvant with BRAF+MEKi | 0 | 3* |
| ***BRAF* V600- Patients** | **Total N=17** | **R** | **NR** |
| SEX | F | 3 | 3 |
| | M | 6 | 5 |
| PRE-THERAPY BIOPSY | | 4 | 5 |
| POST-THERAPY BIOPSY | | 5 | 3 |
| TUMOUR ONSET MEDIAN | | 69.7y | 62.7y |

| | | | |
|---|---|---|---|
| | Anti-PD-1 | 6 | 3 |
| | Anti-CTLA-4 | 1 | 0 |
| THERAPY | Imatinib | 0 | 1 |
| | Adjuvant with Anti-PD-1 | 2 | 3 |
| | Ipilimumab/Adjuvant with Anti-PD-1 | 0 | 1 |

*Abbreviations: F: Female; M: Male; y: years; R: Responder; NR: Non-Responder; *: 1 FFPE biopsy analysed.*

**Supplementary Table S2.** Association between genetic layout and response to therapy.

| VARIABLE | SAMPLES | RESPONSE TO THERAPY | MEDIAN | IQR | Test statistic | p-value |
|---|---|---|---|---|---|---|
| TMB | ALL | R | 22.89 | 10.61-44.28 | W = 197 | p= 0.271 |
| | | NR | 16.04 | 5.3-39 | | |
| | BRAF+ | R | 25.76 | 13.49-51.84 | W = 46 | p= 0.904 |
| | | NR | 34.77 | 7.53-43.97 | | |
| | BRAF- | R | 21.91 | 10.51-44.28 | W = 51 | p=0.167 |
| | | NR | 10.4 | 4.22-24.95 | | |
| | | | | | | |
| N mutations in melanoma driver genes | ALL | R | 2 | 1-3 | W = 211 | p= 0.113 |
| | | NR | 3 | 2-5 | | |
| | BRAF+ | R | 1.5 | 0.75-3.25 | W = 26.5 | p= 0.152 |
| | | NR | 3 | 1.5-5 | | |
| | BRAF- | R | 2 | 1-3 | W = 26 | p= 0.348 |
| | | NR | 3 | 2-3.5 | | |
| | | | | | | |
| N CNVs in melanoma driver genes | ALL | R | 3 | 1-8 | W = 152.5 | p= 0.786 |
| | | NR | 4 | 0.5-11 | | |
| | BRAF+ | R | 3.5 | 1.75-7.5 | W = 40 | p= 0.769 |
| | | NR | 9 | 0-11.5 | | |
| | | | | | | |
| | BRAF- | R | 3 | 1-8 | W = 35 | p= 0.961 |
| | | NR | 3 | 1.75-5.5 | | |
| | | | | | | |
| | | | | | | |
| N DNA Damage Repair genes with LOH | ALL | R | 0 | 0-21 | W = 217 | p= 0.062 |
| | | NR | 12 | 0-35 | | |

| | | | | | | |
|---|---|---|---|---|---|---|
| | BRAF+ | R | 0 | 0-6.75 | W = 57 | p= 0.264 |
| | | NR | 12 | 0-21.5 | | |
| | BRAF- | R | 0 | 0-21 | W = 52 | p= 0.115 |
| | | NR | 20 | 8.25-47 | | |
| | | | | | | |
| COSMIC Signatures | ALL | R | - | - | - | p= 0.754 |
| | | NR | - | - | | |
| | BRAF+ | R | - | - | - | p= 0.699 |
| | | NR | - | - | | |
| | BRAF- | R | - | - | - | p= 0.174 |
| | | NR | - | - | | |
| *TERT* promoter mutations (Yes/No) | ALL | R | - | - | OR = 0.23 (CI = 0.02 - 1.58) | p= 0.118 |
| | | NR | - | - | | |
| | BRAF+ | R | - | - | OR = 0 (CI = 0 - 2.31) | p= 0.234 |
| | | NR | - | - | | |
| | BRAF- | R | - | - | OR = 0.36 (CI = 0.02 - 5.11) | p= 0.580 |
| | | NR | - | - | | |

*Abbreviations*: *N = number of samples; R= responders; NR= non-responders; BRAF+= BRAF-mutated samples; BRAF-= BRAF wild-type samples; W= Wilcoxon rank sum test statistic; OR = odds ratio (in this case, odds that the outcome occurs in R group compared to the odds of the outcome occurring in NR); IQR = inter-quartile range; CI = 95% confidence interval; TMB= tumor mutation burden; LOH= loss of heterozygosity; CNV= copy number variation.*

**Supplementary Table S3.** Loss Of Heterozygosity (LOH) in DNA Damage Repair (DDR) genes for our 36 melanomas patient's cohort.

| Gene | Sample ID | *BRAF* V600 status | R |
|---|---|---|---|
| AEN | #47 | - | n |
| ALKBH1 | #56 | + | n |
| ALKBH3 | #56 | + | n |
| | #4 | - | y |
| | #54 | - | n |
| APEX1 | #56 | + | n |
| APEX2 | #39 | + | y |
| | #19 | + | n |
| | #43 | + | n |
| | #56 | + | n |
| | #1 | + | n |
| | #8 | + | n |
| | #10 | + | n |
| | #50 | + | n |
| | #4 | - | y |
| | #59 | - | y |
| | #32 | - | y |
| | #45 | - | n |
| | #55 | - | n |
| | #63 | - | n |
| | #47 | - | n |
| | #57 | - | n |
| APLF | #47 | - | n |
| APTX | #30 | + | y |
| | #56 | + | n |
| | #10 | + | n |

| | | | |
|---|---|---|---|
| | #59 | - | y |
| | #32 | - | y |
| | #45 | - | n |
| | #54 | - | n |
| | #47 | - | n |
| ASCC3 | #39 | + | y |
| | #10 | + | n |
| | #45 | - | n |
| | #63 | - | n |
| ATM | #39 | + | y |
| | #56 | + | n |
| | #4 | - | y |
| | #54 | - | n |
| ATR | #56 | + | n |
| ATRIP | #56 | + | n |
| ATRX | #39 | + | y |
| | #19 | + | n |
| | #43 | + | n |
| | #56 | + | n |
| | #1 | + | n |
| | #8 | + | n |
| | #10 | + | n |
| | #50 | + | n |
| | #4 | - | y |
| | #59 | - | y |
| | #32 | - | y |
| | #45 | - | n |
| | #55 | - | n |
| | #63 | - | n |
| | #47 | - | n |
| | #57 | - | n |

| | | | |
|---|---|---|---|
| BABAM1 | #59 | - | y |
| BARD1 | #47 | - | n |
| BCAS2 | #56 | + | n |
| | #47 | - | n |
| BLM | #63 | - | n |
| | #47 | - | n |
| BRCA1 | #43 | + | n |
| | #1 | + | n |
| | #8 | + | n |
| | #4 | - | y |
| | #45 | - | n |
| | #54 | - | n |
| BRCA2 | #43 | + | n |
| | #8 | + | n |
| | #10 | + | n |
| BRCC3 | #39 | + | y |
| | #19 | + | n |
| | #43 | + | n |
| | #56 | + | n |
| | #1 | + | n |
| | #8 | + | n |
| | #10 | + | n |
| | #50 | + | n |
| | #4 | - | y |
| | #59 | - | y |
| | #32 | - | y |
| | #45 | - | n |
| | #55 | - | n |
| | #54 | - | n |
| | #63 | - | n |
| | #47 | - | n |

| | | | |
|---|---|---|---|
| | #57 | - | n |
| CCNH | #56 | + | n |
| | #47 | - | n |
| CDC25A | #56 | + | n |
| CDC25C | #47 | - | n |
| CETN2 | #39 | + | y |
| | #19 | + | n |
| | #43 | + | n |
| | #56 | + | n |
| | #1 | + | n |
| | #8 | + | n |
| | #10 | + | n |
| | #50 | + | n |
| | #4 | - | y |
| | #59 | - | y |
| | #32 | - | y |
| | #45 | - | n |
| | #55 | - | n |
| | #54 | - | n |
| | #63 | - | n |
| | #47 | - | n |
| | #57 | - | n |
| CHAF1A | #59 | - | y |
| | #54 | - | n |
| CHEK1 | #39 | + | y |
| | #56 | + | n |
| | #4 | - | y |
| | #59 | - | y |
| | #45 | - | n |
| | #63 | - | n |
| CHEK2 | #47 | - | n |

| | | | |
|---|---|---|---|
| CUL3 | #59 | - | y |
| CUL3 | #47 | - | n |
| CUL5 | #39 | + | y |
| CUL5 | #56 | + | n |
| CUL5 | #4 | - | y |
| CUL5 | #54 | - | n |
| DCLRE1A | #39 | + | y |
| DCLRE1A | #30 | + | y |
| DCLRE1A | #19 | + | n |
| DCLRE1A | #56 | + | n |
| DCLRE1A | #10 | + | n |
| DCLRE1A | #45 | - | n |
| DCLRE1A | #63 | - | n |
| DCLRE1B | #56 | + | n |
| DCLRE1B | #47 | - | n |
| DCLRE1C | #30 | + | y |
| DCLRE1C | #19 | + | n |
| DCLRE1C | #56 | + | n |
| DCLRE1C | #10 | + | n |
| DCLRE1C | #45 | - | n |
| DCLRE1C | #63 | - | n |
| DDB1 | #19 | + | n |
| DDB1 | #56 | + | n |
| DDB1 | #4 | - | y |
| DDB2 | #56 | + | n |
| DDB2 | #4 | - | y |
| DMC1 | #50 | + | n |
| DMC1 | #54 | - | n |
| DMC1 | #47 | - | n |
| DNA2 | #39 | + | y |
| DNA2 | #30 | + | y |

| | | | |
|---|---|---|---|
| | #19 | + | n |
| | #56 | + | n |
| | #10 | + | n |
| | #59 | - | y |
| | #45 | - | n |
| DNTT | #39 | + | y |
| | #30 | + | y |
| | #19 | + | n |
| | #56 | + | n |
| | #10 | + | n |
| | #45 | - | n |
| | #63 | - | n |
| DUT | #39 | + | y |
| | #63 | - | n |
| | #47 | - | n |
| EME1 | #4 | - | y |
| EME2 | #47 | - | n |
| ENDOV | #10 | + | n |
| ERCC2 | #59 | - | y |
| ERCC3 | #47 | - | n |
| ERCC4 | #59 | - | y |
| | #47 | - | n |
| ERCC6 | #39 | + | y |
| | #30 | + | y |
| | #56 | + | n |
| | #10 | + | n |
| | #45 | - | n |
| | #63 | - | n |
| ERCC8 | #57 | - | n |
| EXO1 | #39 | + | y |
| EXO5 | #47 | - | n |

| | | | |
|---|---|---|---|
| FAAP20 | #10 | + | n |
| | #4 | - | y |
| | #47 | - | n |
| FANCA | #39 | + | y |
| | #56 | + | n |
| | #59 | - | y |
| | #45 | - | n |
| | #63 | - | n |
| | #47 | - | n |
| FANCB | #39 | + | y |
| | #19 | + | n |
| | #43 | + | n |
| | #56 | + | n |
| | #1 | + | n |
| | #8 | + | n |
| | #10 | + | n |
| | #50 | + | n |
| | #4 | - | y |
| | #59 | - | y |
| | #32 | - | y |
| | #45 | - | n |
| | #55 | - | n |
| | #54 | - | n |
| | #63 | - | n |
| | #47 | - | n |
| | #57 | - | n |
| FANCC | #30 | + | y |
| | #56 | + | n |
| | #10 | + | n |
| | #32 | - | y |
| | #45 | - | n |

| | | | |
|---|---|---|---|
| | #47 | - | n |
| FANCD2 | #39 | + | y |
| | #56 | + | n |
| FANCE | #56 | + | n |
| FANCF | #56 | + | n |
| | #4 | - | y |
| | #54 | - | n |
| | #63 | - | n |
| FANCG | #30 | + | y |
| | #56 | + | n |
| | #10 | + | n |
| | #32 | - | y |
| | #45 | - | n |
| | #54 | - | n |
| | #47 | - | n |
| FANCI | #47 | - | n |
| FANCL | #56 | + | n |
| | #4 | - | y |
| | #47 | - | n |
| FANCM | #56 | + | n |
| | #59 | - | y |
| FEN1 | #56 | + | n |
| | #4 | - | y |
| GADD45A | #47 | - | n |
| GADD45G | #30 | + | y |
| | #56 | + | n |
| | #10 | + | n |
| | #59 | - | y |
| | #32 | - | y |
| | #45 | - | n |
| | #47 | - | n |

| | | | |
|---|---|---|---|
| gen-01 | #47 | - | n |
| GTF2H1 | #56 | + | n |
| | #4 | - | y |
| | #54 | - | n |
| GTF2H3 | #4 | - | y |
| | #45 | - | n |
| GTF2H5 | #39 | + | y |
| | #56 | + | n |
| | #59 | - | y |
| | #32 | - | y |
| | #45 | - | n |
| | #63 | - | n |
| H2AFX | #39 | + | y |
| | #56 | + | n |
| | #4 | - | y |
| | #59 | - | y |
| | #54 | - | n |
| | #63 | - | n |
| HELQ | #56 | + | n |
| | #63 | - | n |
| HERC2 | #39 | + | y |
| | #63 | - | n |
| | #47 | - | n |
| HES1 | #56 | + | n |
| HFM1 | #47 | - | n |
| HLTF | #56 | + | n |
| HMGB2 | #56 | + | n |
| | #59 | - | y |
| | #63 | - | n |
| HUS1 | #19 | + | n |
| | #54 | - | n |

| | | | |
|---|---|---|---|
| IDH1 | #47 | - | n |
| INO80 | #39 | + | y |
| | #19 | + | n |
| | #63 | - | n |
| | #47 | - | n |
| KAT5 | #56 | + | n |
| | #4 | - | y |
| | #54 | - | n |
| LIG4 | #30 | + | y |
| | #56 | + | n |
| | #57 | - | n |
| MAD2L2 | #10 | + | n |
| | #4 | - | y |
| | #47 | - | n |
| MBD4 | #56 | + | n |
| MGMT | #39 | + | y |
| | #30 | + | y |
| | #19 | + | n |
| | #56 | + | n |
| | #10 | + | n |
| | #45 | - | n |
| | #63 | - | n |
| MLH1 | #56 | + | n |
| MLH3 | #56 | + | n |
| MMS19 | #39 | + | y |
| | #30 | + | y |
| | #19 | + | n |
| | #56 | + | n |
| | #10 | + | n |
| | #63 | - | n |
| MNAT1 | #56 | + | n |

| | | | |
|---|---|---|---|
| MORF4L1 | #47 | - | n |
| MPG | #47 | - | n |
| MRPL40 | #47 | - | n |
| MSH2 | #10 | + | n |
| | #59 | - | y |
| | #47 | - | n |
| MSH6 | #47 | - | n |
| MUS81 | #56 | + | n |
| | #4 | - | y |
| | #54 | - | n |
| MUTYH | #47 | - | n |
| NBN | #4 | - | y |
| | #54 | - | n |
| NEIL1 | #59 | - | y |
| | #47 | - | n |
| NEIL2 | #56 | + | n |
| | #4 | - | y |
| NEIL3 | #56 | + | n |
| | #50 | + | n |
| | #59 | - | y |
| | #63 | - | n |
| NFATC2IP | #47 | - | n |
| NHEJ1 | #47 | - | n |
| NSMCE1 | #59 | - | y |
| | #47 | - | n |
| NSMCE2 | #47 | - | n |
| NSMCE4A | #39 | + | y |
| | #30 | + | y |
| | #19 | + | n |
| | #56 | + | n |
| | #10 | + | n |

| | | | |
|---|---|---|---|
| | #45 | - | n |
| | #63 | - | n |
| NTHL1 | #47 | - | n |
| NUDT1 | #59 | - | y |
| NUDT18 | #56 | + | n |
| | #4 | - | y |
| OGG1 | #39 | + | y |
| | #56 | + | n |
| PALB2 | #47 | - | n |
| PARG | #39 | + | y |
| | #30 | + | y |
| | #56 | + | n |
| | #10 | + | n |
| | #63 | - | n |
| PARP1 | #39 | + | y |
| PARP2 | #56 | + | n |
| PARP3 | #56 | + | n |
| | #54 | - | n |
| PARP4 | #10 | + | n |
| PAXIP1 | #30 | + | y |
| | #56 | + | n |
| PER1 | #19 | + | n |
| | #45 | - | n |
| PLK3 | #47 | - | n |
| PLRG1 | #56 | + | n |
| | #59 | - | y |
| | #63 | - | n |
| PMS1 | #47 | - | n |
| PMS2 | #45 | - | n |
| PNKP | #43 | + | n |
| | #59 | - | y |

| | | | |
|---|---|---|---|
| POLA1 | #39 | + | y |
| | #19 | + | n |
| | #43 | + | n |
| | #56 | + | n |
| | #1 | + | n |
| | #8 | + | n |
| | #10 | + | n |
| | #50 | + | n |
| | #4 | - | y |
| | #59 | - | y |
| | #32 | - | y |
| | #45 | - | n |
| | #55 | - | n |
| | #54 | - | n |
| | #63 | - | n |
| | #47 | - | n |
| | #57 | - | n |
| POLB | #56 | + | n |
| | #4 | - | y |
| POLD3 | #56 | + | n |
| | #4 | - | y |
| POLD4 | #56 | + | n |
| | #4 | - | y |
| | #59 | - | y |
| | #54 | - | n |
| POLE | #8 | + | n |
| | #4 | - | y |
| | #59 | - | y |
| POLE2 | #56 | + | n |
| | #59 | - | y |
| POLE3 | #30 | + | y |

| | | | |
|---|---|---|---|
| | #56 | + | n |
| | #10 | + | n |
| | #32 | - | y |
| | #45 | - | n |
| | #47 | - | n |
| POLE4 | #47 | - | n |
| POLG | #47 | - | n |
| POLI | #54 | - | n |
| POLL | #39 | + | y |
| | #30 | + | y |
| | #19 | + | n |
| | #10 | + | n |
| | #45 | - | n |
| | #63 | - | n |
| POLN | #43 | + | n |
| | #56 | + | n |
| | #59 | - | y |
| | #45 | - | n |
| | #63 | - | n |
| POLQ | #56 | + | n |
| | #50 | + | n |
| PPP4C | #47 | - | n |
| PPP4R1 | #43 | + | n |
| | #59 | - | y |
| PPP4R2 | #56 | + | n |
| PRKDC | #4 | - | y |
| | #63 | - | n |
| PRPF19 | #56 | + | n |
| | #4 | - | y |
| PTEN | #39 | + | y |
| | #30 | + | y |

| | | | |
|---|---|---|---|
| | #56 | + | n |
| | #10 | + | n |
| | #45 | - | n |
| | #63 | - | n |
| RAD18 | #39 | + | y |
| | #56 | + | n |
| RAD23B | #30 | + | y |
| | #56 | + | n |
| | #10 | + | n |
| | #32 | - | y |
| | #45 | - | n |
| | #47 | - | n |
| RAD50 | #47 | - | n |
| RAD51 | #39 | + | y |
| | #19 | + | n |
| | #63 | - | n |
| | #47 | - | n |
| RAD51B | #56 | + | n |
| | #56 | + | n |
| | #45 | - | n |
| RAD52 | #59 | - | y |
| | #63 | - | n |
| RAD54B | #4 | - | y |
| RAD54L | #47 | - | n |
| RAD9A | #56 | + | n |
| | #4 | - | y |
| | #59 | - | y |
| | #54 | - | n |
| RBBP8 | #59 | - | y |
| RBX1 | #47 | - | n |
| RECQL4 | #55 | - | n |

| | | | |
|---|---|---|---|
| | #47 | - | n |
| RECQL5 | #10 | + | n |
| REV1 | #47 | - | n |
| REV3L | #39 | + | y |
| | #10 | + | n |
| | #59 | - | y |
| | #45 | - | n |
| | #63 | - | n |
| RFC1 | #56 | + | n |
| | #63 | - | n |
| RFC2 | #30 | + | y |
| | #56 | + | n |
| RFC3 | #56 | + | n |
| RFC4 | #56 | + | n |
| RIF1 | #39 | + | y |
| | #55 | - | n |
| | #54 | - | n |
| | #47 | - | n |
| RMI1 | #30 | + | y |
| | #56 | + | n |
| | #10 | + | n |
| | #32 | - | y |
| | #45 | - | n |
| | #47 | - | n |
| RMI2 | #47 | - | n |
| RNF168 | #56 | + | n |
| RNF169 | #56 | + | n |
| | #4 | - | y |
| RNF4 | #56 | + | n |
| | #1 | + | n |
| | #63 | - | n |

| | | | |
|---|---|---|---|
| RNMT | #43 | + | n |
| | #59 | - | y |
| RPA1 | #19 | + | n |
| | #45 | - | n |
| RPA2 | #10 | + | n |
| | #47 | - | n |
| RPA4 | #39 | + | y |
| | #19 | + | n |
| | #43 | + | n |
| | #56 | + | n |
| | #1 | + | n |
| | #8 | + | n |
| | #10 | + | n |
| | #50 | + | n |
| | #4 | - | y |
| | #59 | - | y |
| | #32 | - | y |
| | #45 | - | n |
| | #55 | - | n |
| | #63 | - | n |
| | #47 | - | n |
| | #57 | - | n |
| RRM1 | #56 | + | n |
| | #4 | - | y |
| | #45 | - | n |
| RRM2 | #47 | - | n |
| RRM2B | #59 | - | y |
| | #47 | - | n |
| SETMAR | #39 | + | y |
| | #30 | + | y |
| | #56 | + | n |

| | | | |
|---|---|---|---|
| SHPRH | #39 | + | y |
| | #56 | + | n |
| | #32 | - | y |
| | #45 | - | n |
| | #63 | - | n |
| SLX1A | #45 | - | n |
| | #45 | - | n |
| | #47 | - | n |
| SLX4 | #47 | - | n |
| SMARCA4 | #54 | - | n |
| SMARCAD1 | #56 | + | n |
| | #63 | - | n |
| | #47 | - | n |
| SMARCC1 | #56 | + | n |
| SMC5 | #30 | + | y |
| | #56 | + | n |
| | #10 | + | n |
| | #59 | - | y |
| | #32 | - | y |
| | #45 | - | n |
| | #47 | - | n |
| SMC6 | #47 | - | n |
| SOX4 | #56 | + | n |
| SPRTN | #39 | + | y |
| SWI5 | #30 | + | y |
| | #56 | + | n |
| | #10 | + | n |
| | #32 | - | y |
| | #45 | - | n |
| | #47 | - | n |
| SWSAP1 | #54 | - | n |

| | | | |
|---|---|---|---|
| TCEA1 | #4 | - | y |
| | #59 | - | y |
| TDP1 | #56 | + | n |
| TDP2 | #56 | + | n |
| TELO2 | #47 | - | n |
| TOP3A | #19 | + | n |
| TOP3B | #47 | - | n |
| TOPBP1 | #56 | + | n |
| TP53 | #19 | + | n |
| | #45 | - | n |
| TP53BP1 | #39 | + | y |
| | #63 | - | n |
| | #63 | - | n |
| | #47 | - | n |
| TREX1 | #56 | + | n |
| TREX2 | #39 | + | y |
| | #19 | + | n |
| | #43 | + | n |
| | #56 | + | n |
| | #1 | + | n |
| | #8 | + | n |
| | #10 | + | n |
| | #50 | + | n |
| | #4 | - | y |
| | #59 | - | y |
| | #32 | - | y |
| | #45 | - | n |
| | #55 | - | n |
| | #54 | - | n |
| | #63 | - | n |
| | #47 | - | n |

| | | | |
|---|---|---|---|
| | #57 | - | n |
| TTK | #39 | + | y |
| | #10 | + | n |
| | #63 | - | n |
| TYMS | #43 | + | n |
| | #59 | - | y |
| | #45 | - | n |
| UBE2A | #39 | + | y |
| | #19 | + | n |
| | #43 | + | n |
| | #56 | + | n |
| | #1 | + | n |
| | #8 | + | n |
| | #10 | + | n |
| | #50 | + | n |
| | #4 | - | y |
| | #59 | - | y |
| | #32 | - | y |
| | #45 | - | n |
| | #55 | - | n |
| | #63 | - | n |
| | #47 | - | n |
| | #57 | - | n |
| UBE2B | #47 | - | n |
| UBE2T | #39 | + | y |
| UBE2V2 | #4 | - | y |
| | #63 | - | n |
| UIMC1 | #47 | - | n |
| USP1 | #47 | - | n |
| UVSSA | #43 | + | n |
| | #56 | + | n |

| | | | |
|---|---|---|---|
| | #63 | - | n |
| WDR48 | #56 | + | n |
| WEE1 | #56 | + | n |
| | #4 | - | y |
| | #54 | - | n |
| WRN | #56 | + | n |
| | #4 | - | y |
| XAB2 | #54 | - | n |
| XPA | #30 | + | y |
| | #56 | + | n |
| | #10 | + | n |
| | #32 | - | y |
| | #45 | - | n |
| | #47 | - | n |
| XPC | #56 | + | n |
| XRCC2 | #30 | + | y |
| | #56 | + | n |
| | #59 | - | y |
| XRCC3 | #56 | + | n |
| XRCC5 | #47 | - | n |
| XRCC6 | #47 | - | n |
| YWHAG | #30 | + | y |
| ZSWIM7 | #19 | + | n |
| | #45 | - | n |

*Abbreviations: R: Response; y: yes; n: no*

**Supplementary Table S4.** Mutational Signature.

Mutational Signature for our 36 melanomas patient's cohort and comparison between three different tools (DeconstructSigs, SigMa, and SigProfiler v3.2).

| Sample ID | *BRAF* V600 Status | deconstructSig | SigMa | ssSigProfiler |
|---|---|---|---|---|
| #1 | + | Signature_3 | Signature_3_hc | SBS3 |
| #2 | + | Signature_3 | Signature_3_hc | SBS3 |
| #3 | + | Signature_3 | Signature_3_hc | SBS3 |
| #8 | + | Signature_3 | Signature_3_hc | SBS3 |
| #10 | + | Signature_3 | Signature_3_hc | SBS3 |
| #14 | + | Signature_3 | Signature_3_hc | SBS3 |
| #17 | + | Signature_clock-like | Signature_clock | SBS5 |
| #19 | + | Signature_UV | Signature_UV | SBS7 |
| #26 | + | Signature_clock-like | Signature_clock | SBS5 |
| #30 | + | Signature_UV | Signature_UV | SBS7 |
| #37 | + | Signature_clock-like | Signature_clock | SBS5 |
| #39 | + | Signature_UV | Signature_UV | SBS7 |
| #40 | + | Signature_UV | Signature_UV | SBS7 |
| #42 | + | Signature_clock-like | Signature_3_hc | SBS3 |
| #43 | + | Signature_clock-like | Signature_msi | SBS1 |
| #50 | + | Signature_clock-like | Signature_clock | SBS5 |
| #56 | + | Signature_clock-like | Signature_clock | SBS5 |

| | | | | |
|---|---|---|---|---|
| #60 | + | Signature_UV | Signature_UV | SBS7 |
| #62 | + | Signature_UV | Signature_UV | SBS7 |
| #4 | - | Signature_UV | Signature_UV | SBS7 |
| #6 | - | Signature_3 | Signature_3_hc | SBS3 |
| #7 | - | Signature_UV | Signature_UV | SBS7 |
| #18 | - | Signature_UV | Signature_UV | SBS7 |
| #20 | - | Signature_UV | Signature_UV | SBS7 |
| #21 | - | Signature_3 | Signature_3_hc | SBS3 |
| #32 | - | Signature_UV | Signature_UV | SBS7 |
| #34 | - | Signature_3 | Signature_3_hc | SBS3 |
| #38 | - | Signature_UV | Signature_UV | SBS7 |
| #45 | - | Signature_UV | Signature_UV | SBS7 |
| #46 | - | Signature_3 | Signature_3_hc | SBS3 |
| #47 | - | Signature_clock-like | Signature_clock | SBS5 |
| #54 | - | Signature_clock-like | Signature_clock | SBS5 |
| #55 | - | Signature_clock-like | Signature_UV | SBS7 |
| #57 | - | Signature_UV | Signature_UV | SBS7 |
| #59 | - | Signature_UV | Signature_UV | SBS7 |
| #63 | - | Signature_clock-like | Signature_clock | SBS5 |

*Abbreviations: +: positive; -: negative*

**Supplementary Table S5.** Copy Number Variations (CNVs) in melanoma driver genes detected by Whole-Exome Sequencing (WES) in common between the pre-therapy and post-therapy melanoma lesions.

| Gene | CNV | Sample ID | *BRAF* V600 status | R |
|---|---|---|---|---|
| | | | | |
| *PTEN* | DELETION | #39 | + | y |
| | | #34 | - | n |
| | | #63 | - | n |
| *FBXW7* | DELETION | #63 | - | n |
| *KIT* | DELETION | #63 | - | n |

| | | | | |
|---|---|---|---|---|
| *DDX3X* | DELETION | #34 | - | n |
| | | #63 | - | n |
| *BRAF* | AMPLIFICATION | #18 | - | n |
| *KIT* | AMPLIFICATION | #21 | - | y |
| | | #34 | - | n |
| *STK19* | AMPLIFICATION | #21 | - | y |
| | | #7 | - | y |
| | | #34 | - | n |

| | | | | |
|---|---|---|---|---|
| | | #21 | - | y |
| *EZH2* | AMPLIFICATION | #34 | - | n |
| | | #18 | - | n |
| *NRAS* | AMPLIFICATION | #34 | - | n |
| *MAP2K2* | AMPLIFICATION | #34 | - | n |
| *RAC1* | AMPLIFICATION | #34 | - | n |
| *SNX31* | AMPLIFICATION | #34 | - | n |
| *TACC1* | AMPLIFICATION | #34 | - | n |

| | | | | |
|---|---|---|---|---|
| *TERT* | AMPLIFICATION | #34 | - | n |
| *GNA11* | AMPLIFICATION | #34 | - | n |

*Abbreviations: R: Response; n: no; y: yes: -: Negative; +: Positive; CNV: Copy Number Variation.*

**Supplementary Table S6.** Copy Number Variations (CNVs) in melanoma driver genes detected by Whole-Exome Sequencing (WES) acquired in the post-therapy melanoma lesions.

| Gene | CNV | Sample ID | *BRAF* V600 status | R |
|---|---|---|---|---|
| | | #39§ | + | y |
| *BRAF* | AMPLIFICATION | #1§ | + | n |
| | | #20 | - | y |
| | | #1 | + | n |
| *HRAS* | AMPLIFICATION | #20 | - | y |
| | | #34 | - | n |
| *CDK4* | AMPLIFICATION | #1 | + | n |

| | | | | |
|---|---|---|---|---|
| *KRAS* | AMPLIFICATION | #18 | - | n |
| *MAP2K1* | AMPLIFICATION | #20 | - | y |
| *MAP2K2* | AMPLIFICATION | #42 | + | y |
| | | #1 | + | n |
| *GNA11* | AMPLIFICATION | #1 | + | n |
| | | #42 | + | y |
| | | #20 | - | y |
| *EZH2* | AMPLIFICATION | #42 | + | y |

| | | | | |
|---|---|---|---|---|
| | | #20 | - | y |
| | | #57 | - | n |
| *FBXW7* | AMPLIFICATION | #21 | - | y |
| | | #1 | + | n |
| *MTOR* | AMPLIFICATION | #42 | + | y |
| | | #20 | - | y |
| *NRAS* | AMPLIFICATION | #18 | - | n |
| | | #57 | - | n |

| | | | | |
|---|---|---|---|---|
| *PPP6C* | AMPLIFICATION | #1 | + | n |
| | | #20 | - | y |
| *RAC1* | AMPLIFICATION | #1 | + | n |
| | | #42 | + | y |
| | | #20 | - | y |
| *RB1* | AMPLIFICATION | #1 | + | n |
| *STK19* | AMPLIFICATION | #1 | + | n |
| | | #42 | + | y |

| | | | | |
|---|---|---|---|---|
| *TERT* | AMPLIFICATION | #20 | - | y |
| *TP53* | AMPLIFICATION | #20 | - | y |
| *WT1* | AMPLIFICATION | #20 | - | y |
| *FBXW7* | DELETION | #20 | - | y |
| *CDKN2A* | DELETION | #20 | - | y |
| *CNOT9* | DELETION | #20 | - | y |
| *CTNNB1* | DELETION | #20 | - | y |
| *DDX3X* | DELETION | #1 | + | n |

| | | | | |
|---|---|---|---|---|
| | | #20 | - | y |
| *GNA11* | DELETION | #63 | - | n |
| | | #57 | - | n |
| *HRAS* | DELETION | #42 | + | y |
| *IDH1* | DELETION | #20 | - | y |
| *MAP2K1* | DELETION | #20 | - | y |
| *MAP2K2* | DELETION | #63 | - | n |
| | | #57 | - | n |

| | | | | |
|---|---|---|---|---|
| *TACC1* | DELETION | #20 | - | y |
| *TP53* | DELETION | #63 | - | n |

*Abbreviations: R: Response; n: no; y: yes; -: Negative; +: Positive; CNV: Copy number variation; §: gain present in the pre-lesions and post lesion.*

**Supplementary Table S7.** Loss Of Function (LOF) variants in DNA Damage Repair (DDR) genes.

For each of 36 patients, exonic somatic LOF variants in DDR genes with an Allele Frequency (AF) at least 10% are reported. In bold the variants found in common in the two lesions of the same patient.

| Chr | *BRAF* V600 status | R | Gene | aa change | Ref seq | Coverage | AF |
|---|---|---|---|---|---|---|---|
| #2 | + | y | *TP53BP1* | p.Ser1359fs | *NM_001141980.3* | *58* | *63.6* |
| #2 | + | y | *DDB1* | p.Arg989fs | *NM_001923.5* | *45* | *42.9* |
| #17 | + | y | *RAD51B* | p.Gln371* | *NM_133509.4* | *76* | *20.0* |
| #39_PRE | + | y | *TDG* | p.Ile98fs | *NM_003211.6* | *195* | *20.0* |
| #39_PRE | + | y | *CUL4B* | p.Ser61fs | *NM_003588.3* | *63* | *12.8* |
| #39 | + | y | *CUL5* | p.Gln709* | *NM_003478.6* | *965* | *59.6* |
| #40 | + | y | *MSH6* | p.Asp1026fs | *NM_000179.2* | *585* | *10.1* |
| #42 | + | y | *ERCC6* | p.Ala298delinsGlyPheSerSerTerSerValProSer | *NM_000124.4* | *61* | *45.5* |

| | | | | | | | |
|---|---|---|---|---|---|---|---|
| #42 | + | y | *MLH3* | p.Asn629fs | *NM_001040108.1* | *182* | *11.4* |
| #10 | + | n | *MDC1* | p.Arg944fs | *NM_014641.3.5* | *202* | *12.5* |
| #10 | + | n | *TP53BP1* | p.Ser1359fs | *NM_001141980.3* | *88* | *50.0* |
| #10 | + | n | *POLM* | p.Arg248* | *NM_001284330.2* | *1488* | *30.3* |
| #10 | + | n | *ERCC6* | p.Ala298delinsGlyPheSerSerTerSerValProSer | *NM_000124.4* | *85* | *66.7* |
| #14 | + | n | *HERC2* | p.Gln142fs | *NM_004667.5* | *67* | *17.6* |
| #14 | + | n | *TP53BP1* | p.Ser1359fs | *NM_001141980.3* | *83* | *20.0* |
| #14 | + | n | *ERCC6* | p.Ala298delinsGlyPheSerSerTerSerValProSer | *NM_000124.4* | *92* | *26.9* |
| #14 | + | n | *LIG4* | p.Thr554fs | *NM_001352604.1* | *173* | *23.0* |
| #14 | + | n | *SHPRH* | p.Asp944fs | *NM_001042683.3* | *38* | *21.4* |

| | | | | | | | |
|---|---|---|---|---|---|---|---|
| #19 | + | n | *PTEN* | p.Arg303fs | *NM_001085384.3* | *410* | *64.8* |
| #3 | + | n | *CHEK2* | p.Ser499fs | *NM_001005735.2* | *83* | *23.5* |
| #3 | + | n | *FANCE* | p.Arg176fs | *NM_021922.3* | *81* | *14.6* |
| #3 | + | n | *TP53BP1* | p.Ser1359fs | *NM_001141980.3* | *60* | *25.0* |
| #3 | + | n | *ERCC6* | p.Ala298delinsGlyPheSerSerTerSerValProSer | *NM_000124.4* | *71* | *37.5* |
| #3 | + | n | *APEX2* | p.Thr295fs | *NM_014481* | *88* | *23.8* |
| #3 | + | n | *FANCA* | p.Ala1215fs | *NM_000135.4* | *81* | *18.9* |
| #3 | + | n | *TDP1* | p.Asp346fs | *NM_001008744.2* | *59* | *53.8* |
| #3 | + | n | *SHPRH* | p.Glu131* | *NM_001042683.3* | *117* | *15.2* |
| #62_POST | + | n | *GEN1* | p.Lys839fs | *NM_001130009.3* | *368* | *45.0* |

| | | | | | | | |
|---|---|---|---|---|---|---|---|
| #62_POST | + | n | *PMS1* | p.Leu164fs | *NM_001321049.2* | *269* | *44.1* |
| #6 | - | y | *RFC1* | p.Thr132fs | *NM_001204747.2* | *190* | *14.9* |
| #6 | - | y | *TP53BP1* | p.Ser980_Gly981insIleArgGly | *NM_001141980.3* | *36* | *33.3* |
| #6 | - | y | *ERCC6* | p.Ala298delinsGlyPheSerSerTerSerValProSer | *NM_000124.4* | *141* | *23.1* |
| #6 | - | y | *APEX2* | p.Thr295fs | *NM_014481* | *88* | *15.6* |
| #6 | - | y | *RBP1* | p.Ter158Glnext*? | *NM_001130992.2* | *45* | *18.2* |
| #20_PRE | - | y | *APEX2* | p.Thr295fs | *NM_014481* | *51* | *50.0* |
| #20_PRE | - | y | *ERCC6* | p.Ala298delinsGlyPheSerSerTerSerValProSer | *NM_000124.4* | *164* | *21.2* |
| #20_POST | - | y | *ERCC6* | p.Ala298delinsGlyPheSerSerTerSerValProSer | *NM_000124.4* | *115* | *41.2* |
| #20_POST | - | y | *LIG4* | p.Thr554fs | *NM_001352604.1* | *280* | *13.3* |

| | | | | | | | |
|---|---|---|---|---|---|---|---|
| #20_POST | - | y | *REV3L* | p.Lys1044delinsAsnLeuTrpAspLeuCysPheSerGlnLysGluAsnLeuValTerThrTerHisHisTyrAsnSer | *NM_001372078.1* | *101* | *23.1* |
| #20_POST | - | y | *DCLRE1B* | p.Lys290_Pro291insIleProValGlyProSerThrTerSerProSerPheGluThrHisArgLysAlaLeu | *NM_022836.4* | *115* | *20.0* |
| #20_POST | - | y | TP53BP1 | p.Thr514fs | NM_001141980.3 | 129 | 16.7 |
| #21 | - | y | *GTF2H1* | p.Trp33* | *NM_001142307.1* | *109* | *12.0* |
| #21 | - | y | *TDP2* | p.Thr230fs | *NM_016614.3* | *103* | *33.3* |
| #21 | - | y | *BRCA1* | p.Ser377fs | *NM_007300.4* | *254* | *27.5* |
| #21 | - | y | *TP53BP1* | p.Ser1359fs | *NM_001141980.3* | *138* | *21.7* |
| #21 | - | y | *ATRX* | p.Ala1410fs | *NM_000489.5* | *89* | *23.5* |
| #21 | - | y | *RAD54B* | p.Lys132* | *NM_012415.3* | *279* | *18.8* |
| #38 | - | y | *BRCA2* | p.Glu954* | *NM_000059.3* | *376* | *34.5* |

| | | | | | | | |
|---|---|---|---|---|---|---|---|
| #46 | - | y | *ERCC6* | p.Ala298delinsGlyPheSerSerTerSerValProSer | *NM_000124.4* | *78* | *37.5* |
| #59 | - | y | *BLM* | p.Gln83* | *NM_000057.4* | *688* | *11.1* |
| #34_PRE | - | n | *TDP2* | p.Thr230fs | *NM_016614.3* | *43* | *29.4* |
| #34_PRE | - | n | *MDC1* | p.Val440_Leu441insAlaHisProGlyProProProHisGluLeuSerProAlaTerGluGlnLeuGlnProThrLeuProGlnHisPro | *NM_014641.3.5* | *59* | *23.5* |
| #34_PRE | - | n | *BRCA1* | p.Ser377fs | *NM_007300.4* | *172* | *30.3* |
| #34_PRE | - | n | *TP53BP1* | **p.Ser1359fs** | *NM_001141980.3* | *68* | *26.9* |
| #34 | - | n | *TP53BP1* | **p.Ser1359fs** | *NM_001141980.3* | *66* | *15.4* |
| #34_PRE | - | n | *CDC5L* | p.Gly604fs | *NM_001253.4* | *159* | *10.3* |
| #34_PRE | - | n | *ERCC6* | **p.Ala298delinsGlyPheSerSerTerSerValProSer** | *NM_000124.4* | *79* | *80.0* |
| #34 | - | n | *ERCC6* | **p.Ala298delinsGlyPheSerSerTerSerValProSer** | *NM_000124.4* | *100* | *25.9* |

| | | | | | | | |
|---|---|---|---|---|---|---|---|
| #34_PRE | - | n | *REV1* | p.Leu289fs | *NM_001321454.2* | *51* | *23.1* |
| #34_PRE | - | n | *LIG4* | **p.Thr554fs** | *NM_001352604.1* | *182* | *15.6* |
| #34 | - | n | *LIG4* | **p.Thr554fs** | *NM_001352604.1* | *204* | *11.2* |
| #34 | - | n | *FANCE* | p.Arg176fs | *NM_021922.3* | *95* | *17.2* |
| #34 | - | n | *MLH3* | p.Asn629fs | *NM_001040108.1* | *218* | *11.8* |
| #34 | - | n | *ATR* | p.Val2230fs | *NM_001184.4* | *68* | *21.9* |
| #63_PRE | - | n | *ERCC6* | **p.Arg1221*** | *NM_000124.4* | *329* | *64.6* |
| #63 | - | n | *ERCC6* | **p.Arg1221*** | *NM_000124.4* | *340* | *65.4* |

*Abbreviations: -:Negative; +: Positive; y: Yes; n: No; AF: Allele Frequency.*

**Supplementary Table S8.** Loss of heterozygosity (LOH)  in in DNA Damage Repair (DDR) genes detected by Whole-Exome Sequencing (WES) in common between the pre-therapy and post-therapy melanoma lesions.

| Gene | Sample ID | *BRAF* V600 status | R |
|---|---|---|---|
| APEX2 | #39 | + | y |
| | #63 | - | n |
| ATRX | #39 | + | y |
| | #63 | - | n |
| BRCC3 | #39 | + | y |
| | #63 | - | n |
| CETN2 | #39 | + | y |
| | #63 | - | n |
| FANCB | #39 | + | y |
| | #63 | - | n |
| POLA1 | #39 | + | y |
| | #63 | - | n |
| RPA4 | #39 | + | y |
| | #63 | - | n |
| TREX2 | #39 | + | y |
| | #63 | - | n |
| UBE2A | #39 | + | y |
| | #63 | - | n |
| ASCC3 | #63 | - | n |
| ATM | #63 | - | n |
| CHEK1 | #63 | - | n |
| DCLRE1A | #63 | - | n |
| DCLRE1C | #63 | - | n |
| DNTT | #63 | - | n |
| DUT | #63 | - | n |

| | | | |
|---|---|---|---|
| ERCC6 | #63 | - | n |
| FANCA | #63 | - | n |
| GTF2H5 | #63 | - | n |
| H2AFX | #63 | - | n |
| HELQ | #63 | - | n |
| HERC2 | #63 | - | n |
| HMGB2 | #63 | - | n |
| INO80 | #63 | - | n |
| MGMT | #63 | - | n |
| MMS19 | #63 | - | n |
| NEIL3 | #63 | - | n |
| NSMCE4A | #63 | - | n |
| PARG | #63 | - | n |
| PLRG1 | #63 | - | n |
| POLL | #63 | - | n |
| POLN | #63 | - | n |
| PTEN | #63 | - | n |
| RAD51 | #63 | - | n |
| REV3L | #63 | - | n |
| RFC1 | #63 | - | n |
| RNF4 | #63 | - | n |
| SHPRH | #63 | - | n |
| SMARCAD1 | #63 | - | n |
| TP53BP1 | #63 | - | n |
| TTK | #63 | - | n |
| UVSSA | #63 | - | n |

*Abbreviations: R: Response; Y: yes; N: no*

**Supplementary Table S9.** Loss of heterozygosity (LOH) in DNA Damage Repair (DDR) genes detected by Whole-Exome Sequencing (WES) acquired in the post-therapy melanoma lesions.

| Gene | Sample ID | *BRAF* V600 status | R |
|---|---|---|---|
| APEX2 | #1 | + | n |
| | #62 | + | n |
| | #21 | - | y |
| APTX | #62 | + | n |
| | #21 | - | y |
| ASCC3 | #39 | + | y |
| | #62 | + | n |
| | #21 | - | y |
| ATM | #39 | + | y |
| | #62 | + | n |
| ATRX | #1 | + | n |
| | #62 | + | n |
| | #21 | - | y |
| BLM | #62 | + | n |
| | #63 | - | n |
| BRCA1 | #1 | + | n |
| | #62 | + | n |
| | #21 | - | y |
| BRCC3 | #1 | + | n |
| | #62 | + | n |
| | #21 | - | y |
| BRIP1 | #62 | + | n |
| | #21 | - | y |
| CDC5L | #62 | + | n |
| | #21 | - | y |
| CETN2 | #1 | + | n |

| | | | |
|---|---|---|---|
| | #62 | + | n |
| | #21 | - | y |
| CHEK1 | #39 | + | y |
| | #62 | + | n |
| | #62 | + | n |
| CHEK2 | #62 | + | n |
| | #21 | - | y |
| CUL5 | #39 | + | y |
| | #62 | + | n |
| DCLRE1A | #39 | + | y |
| | #62 | + | n |
| DMC1 | #62 | + | n |
| | #21 | - | y |
| DNA2 | #39 | + | y |
| | #62 | + | n |
| DUT | #39 | + | y |
| | #62 | + | n |
| ENDOV | #62 | + | n |
| | #21 | - | y |
| ERCC6 | #39 | + | y |
| | #62 | + | n |
| FAAP100 | #62 | + | n |
| | #21 | - | y |
| FANCB | #1 | + | n |
| | #62 | + | n |
| | #21 | - | y |
| FANCC | #62 | + | n |
| | #21 | - | y |
| FANCD2 | #39 | + | y |
| | #62 | + | n |
| FANCE | #62 | + | n |
| | #21 | - | y |

| | | | |
|---|---|---|---|
| FANCF | #62 | + | n |
| | #63 | - | n |
| FANCG | #62 | + | n |
| | #21 | - | y |
| GADD45G | #62 | + | n |
| | #21 | - | y |
| GTF2H5 | #39 | + | y |
| | #62 | + | n |
| | #21 | - | y |
| H2AFX | #39 | + | y |
| | #62 | + | n |
| HERC2 | #39 | + | y |
| | #62 | + | n |
| HUS1 | #62 | + | n |
| | #62 | + | n |
| INO80 | #39 | + | y |
| | #62 | + | n |
| MMS19 | #39 | + | y |
| | #62 | + | n |
| NSMCE4A | #39 | + | y |
| | #62 | + | n |
| OGG1 | #39 | + | y |
| | #62 | + | n |
| PARG | #39 | + | y |
| | #62 | + | n |
| PARP1 | #39 | + | y |
| | #62 | + | n |
| POLA1 | #1 | + | n |
| | #62 | + | n |
| | #21 | - | y |
| POLE3 | #62 | + | n |
| | #21 | - | y |

| | | | |
|---|---|---|---|
| POLH | #62 | + | n |
| | #21 | - | y |
| POLL | #39 | + | y |
| | #62 | + | n |
| PRKDC | #62 | + | n |
| | #63 | - | n |
| RAD18 | #39 | + | y |
| | #62 | + | n |
| RAD23B | #62 | + | n |
| | #21 | - | y |
| RAD51C | #62 | + | n |
| | #21 | - | y |
| RAD52 | #62 | + | n |
| | #63 | - | n |
| RBX1 | #62 | + | n |
| | #21 | - | y |
| REV3L | #39 | + | y |
| | #62 | + | n |
| | #21 | - | y |
| RMI1 | #62 | + | n |
| | #21 | - | y |
| RNF4 | #1 | + | n |
| | #62 | + | n |
| RNF8 | #62 | + | n |
| | #21 | - | y |
| RPA4 | #1 | + | n |
| | #62 | + | n |
| | #21 | - | y |
| SETMAR | #39 | + | y |
| | #62 | + | n |
| SHPRH | #39 | + | y |
| | #62 | + | n |

| | | | |
|---|---|---|---|
| | #21 | - | y |
| SMC5 | #62 | + | n |
| | #21 | - | y |
| SOX4 | #62 | + | n |
| | #21 | - | y |
| SPRTN | #39 | + | y |
| | #62 | + | n |
| SWI5 | #62 | + | n |
| | #21 | - | y |
| TDP2 | #62 | + | n |
| | #21 | - | y |
| TOP3B | #62 | + | n |
| | #21 | - | y |
| TP53BP1 | #39 | + | y |
| | #62 | + | n |
| TREX2 | #1 | + | n |
| | #62 | + | n |
| | #21 | - | y |
| TTK | #39 | + | y |
| | #62 | + | n |
| | #21 | - | y |
| UBE2A | #1 | + | n |
| | #62 | + | n |
| | #21 | - | y |
| UBE2T | #39 | + | y |
| | #62 | + | n |
| UBE2V2 | #62 | + | n |
| | #63 | - | n |
| XRCC6 | #62 | + | n |
| | #21 | - | y |
| AEN | #62 | + | n |
| ALKBH1 | #62 | + | n |

| | | | |
|---|---|---|---|
| ALKBH2 | #62 | + | n |
| ALKBH3 | #62 | + | n |
| APEX1 | #62 | + | n |
| APLF | #62 | + | n |
| ATR | #62 | + | n |
| BABAM1 | #62 | + | n |
| BARD1 | #62 | + | n |
| BCAS2 | #62 | + | n |
| BRCA2 | #62 | + | n |
| CCNH | #62 | + | n |
| CDC25B | #62 | + | n |
| CDC25C | #62 | + | n |
| CHAF1A | #62 | + | n |
| CUL3 | #62 | + | n |
| DCLRE1C | #62 | + | n |
| DDB1 | #62 | + | n |
| DDB2 | #62 | + | n |
| DNTT | #39 | + | y |
| EID3 | #62 | + | n |
| EME1 | #21 | - | y |
| EME2 | #62 | + | n |
| ERCC1 | #62 | + | n |
| ERCC2 | #62 | + | n |
| ERCC4 | #62 | + | n |
| ERCC5 | #62 | + | n |
| ERCC8 | #62 | + | n |
| EXO1 | #39 | + | y |
| EXO5 | #62 | + | n |
| FAAP20 | #62 | + | n |
| FAAP24 | #62 | + | n |
| FANCA | #39 | + | y |
| FANCI | #62 | + | n |

| | | | |
|---|---|---|---|
| FANCM | #62 | + | n |
| FEN1 | #62 | + | n |
| GADD45A | #62 | + | n |
| GEN1 | #62 | + | n |
| GTF2H3 | #62 | + | n |
| HELQ | #62 | + | n |
| HES1 | #62 | + | n |
| HFM1 | #62 | + | n |
| HMGB1 | #62 | + | n |
| HMGB2 | #62 | + | n |
| IDH1 | #62 | + | n |
| LIG1 | #62 | + | n |
| LIG3 | #62 | + | n |
| LIG4 | #62 | + | n |
| MAD2L2 | #62 | + | n |
| MBD4 | #62 | + | n |
| MGMT | #39 | + | y |
| MLH1 | #62 | + | n |
| MLH3 | #62 | + | n |
| MNAT1 | #62 | + | n |
| MORF4L1 | #62 | + | n |
| MPG | #62 | + | n |
| MPLKIP | #62 | + | n |
| MRPL40 | #21 | - | y |
| MSH2 | #62 | + | n |
| MSH3 | #62 | + | n |
| MSH6 | #62 | + | n |
| NABP2 | #62 | + | n |
| NBN | #62 | + | n |
| NEIL2 | #62 | + | n |
| NEIL3 | #62 | + | n |
| NFATC2IP | #62 | + | n |

| | | | |
|---|---|---|---|
| NHEJ1 | #62 | + | n |
| NSMCE1 | #62 | + | n |
| NTHL1 | #62 | + | n |
| NUDT15 | #62 | + | n |
| NUDT18 | #62 | + | n |
| PALB2 | #62 | + | n |
| PARP2 | #62 | + | n |
| PARP3 | #62 | + | n |
| PARP4 | #62 | + | n |
| PARPBP | #62 | + | n |
| PAXIP1 | #62 | + | n |
| PER1 | #62 | + | n |
| PLK3 | #62 | + | n |
| PLRG1 | #62 | + | n |
| PMS1 | #62 | + | n |
| PMS2 | #62 | + | n |
| POLB | #62 | + | n |
| POLD1 | #62 | + | n |
| POLD2 | #62 | + | n |
| POLD3 | #62 | + | n |
| POLD4 | #62 | + | n |
| POLE | #62 | + | n |
| POLE2 | #62 | + | n |
| POLE4 | #62 | + | n |
| POLG | #62 | + | n |
| POLI | #62 | + | n |
| POLM | #62 | + | n |
| POLQ | #62 | + | n |
| PPP4C | #62 | + | n |
| PPP4R1 | #62 | + | n |
| PPP4R2 | #62 | + | n |
| PRPF19 | #62 | + | n |

| | | | |
|---|---|---|---|
| PTEN | #39 | + | y |
| RAD1 | #62 | + | n |
| RAD50 | #62 | + | n |
| RAD51 | #39 | + | y |
| RAD51B | #62 | + | n |
| RAD51D | #62 | + | n |
| RAD54B | #62 | + | n |
| RAD9B | #62 | + | n |
| RECQL4 | #62 | + | n |
| RECQL5 | #21 | - | y |
| REV1 | #62 | + | n |
| RFC1 | #62 | + | n |
| RFC2 | #62 | + | n |
| RFC3 | #62 | + | n |
| RFC4 | #62 | + | n |
| RFC5 | #62 | + | n |
| RIF1 | #39 | + | y |
| RMI2 | #62 | + | n |
| RNF168 | #62 | + | n |
| RNF169 | #62 | + | n |
| RNMT | #62 | + | n |
| RPA1 | #62 | + | n |
| RPA2 | #62 | + | n |
| RPA3 | #62 | + | n |
| RRM1 | #62 | + | n |
| RRM2 | #62 | + | n |
| RRM2B | #62 | + | n |
| RTEL1 | #62 | + | n |
| SLX1A | #62 | + | n |
| SLX4 | #62 | + | n |
| SMARCAD1 | #62 | + | n |
| SMC6 | #62 | + | n |

| | | | |
|---|---|---|---|
| SMUG1 | #62 | + | n |
| SPO11 | #62 | + | n |
| TCEA1 | #62 | + | n |
| TDG | #62 | + | n |
| TDP1 | #62 | + | n |
| TELO2 | #62 | + | n |
| TOP3A | #62 | + | n |
| TOPBP1 | #62 | + | n |
| TP53 | #62 | + | n |
| TYMS | #62 | + | n |
| UBE2B | #62 | + | n |
| UBE2N | #62 | + | n |
| UIMC1 | #62 | + | n |
| UNG | #62 | + | n |
| USP1 | #62 | + | n |
| WDR48 | #62 | + | n |
| WRN | #62 | + | n |
| XAB2 | #62 | + | n |
| XPA | #21 | - | y |
| XPC | #62 | + | n |
| XRCC1 | #62 | + | n |
| XRCC2 | #62 | + | n |
| XRCC3 | #62 | + | n |
| XRCC5 | #62 | + | n |
| YWHAB | #62 | + | n |
| YWHAG | #62 | + | n |
| ZSWIM7 | #62 | + | n |

*Abbreviations: R: Response; y: yes; n: no*

**Supplementary Table S10**. Circulating free DNA (cfDNA) mutational profile.

| Sample ID | Sex | R | Stage | *BRAF* V600 status | Previous treatment | Treatment | cfDNA-NGS results | AF% | Tissue-WES results | AF% | Time variation from tissue to cfDNA (days) | Time variation from therapy to cfDNA (days) |
|---|---|---|---|---|---|---|---|---|---|---|---|---|
| #2 | M | Y | IV | + | Y | BRAF+MEKi | *BRAF* p.Val600Glu | 4.8 | *BRAF* p.Val600Glu | 52.4 | 15 | 369 |
| | | | | | | | *NRAS* p.Gln61Lys | 5.0 | - | - | | |
| | | | | | | | *CDK4* Amplification | 1.3 | *CDK4* Amplification | 4 copies | | |
| | | | | | | | - | - | *CCND3* amplification | 3 copies | | |
| | | | | | | | - | - | *CCND1* amplification | 3 copies | | |
| | | | | | | | - | - | *EGFR* amplification | 3 copies | | |
| | | | | | | | - | - | *FGFR3* amplification | 4 copies | | |
| | | | | | | | - | - | *FGFR2* deletion | 1copy | | |
| #17 | F | Y | IV | + | Y | ICB | *BRAF* p.Val600Glu | 0.3 | *BRAF* p.Val600Glu | 42.0 | 610 | 946 |
| | | | | | | | - | - | *FGFR3* amplification | 4 copies | | |
| | | | | | | | - | - | *CCND1* amplification | 3 copies | | |
| #26 | M | Y | IV | + | y | BRAF+MEKi | - | - | *BRAF* p.Val600Glu | 8.0 | 39 | 482 |
| | | | | | | | - | - | *FGFR3* amplification | 3 copies | | |
| #39 | M | Y | IV | + | Y | BRAF+MEKi and ICB | *BRAF* p.Val600Glu | 32.6 | *BRAF* p.Val600Glu | 97.0 | 137 | 1329 |
| | | | | | | | - | - | *FGFR2* deletion | 1 copy | | |
| #1 | M | N | IV | + | Y | BRAF+MEKi | *BRAF* p.Val600Glu | 67.7 | *BRAF* p.Val600Glu | 77.0 | 19 | 245 |
| | | | | | | | *NRAS* p.Gln61Lys | 3.5 | - | - | | |
| | | | | | | | *MAP2K2* p.Gln60Pro | 0.9 | - | - | | |
| | | | | | | | *MET* Amplification | 4.3 | - | - | | |
| | | | | | | | - | - | *CCND1* amplification | 3 copies | | |
| | | | | | | | - | - | *CCND3* amplification | 3 copies | | |
| | | | | | | | - | - | *CDK4* amplification | 4 copies | | |
| | | | | | | | - | - | *FGFR3* amplification | 4 copies | | |
| #3 | F | N | IV | + | N | - | - | - | *BRAF* p.Val600Glu | 16.7 | 16 | 0 |

| | | | | | | | | | | | | |
|---|---|---|---|---|---|---|---|---|---|---|---|
| | | | | | | | *SMO* p.Pro641Ala | 50 | - | - | | |
| | | | | | | | - | - | *CCND1* amplification | 3 copies | | |
| | | | | | | | - | - | *CDK4* amplification | 3 copies | | |
| | | | | | | | - | - | *FGFR3* amplification | 5 copies | | |
| | | | | | | | - | - | *EGFR* amplification | 3 copies | | |
| #8_T1 | M | N | IV | + | N | - | - | - | *BRAF* p.Val600Glu | 22 | 14 | 0 |
| | | | | | | | - | - | *CCND1* amplification | 3 copies | | |
| | | | | | | | - | - | *EGFR* amplification | 3 copies | | |
| | | | | | | | - | - | *FGFR3* amplification | 3 copies | | |
| #8_T2 | M | N | IV | + | Y | BRAF+MEKi | *BRAF* p.Val600Glu | 3.8 | *BRAF* p.Val600Glu | 22 | 271 | 257 |
| | | | | | | | *BRAF* p.Val600Met | 0.8 | - | - | | |
| | | | | | | | *PDGFRA* p.Ala633Thr | 0.24 | - | - | | |
| #10 | F | N | IV | + | Y | BRAF+MEKi and ICB | *BRAF* p.Val600Glu | 8.8 | *BRAF* p.Val600Glu | 60.0 | 289 | 461 |
| | | | | | | | *GNA11* p.Gln209Leu | 3.7 | - | - | | |
| | | | | | | | - | - | *FGFR3* amplification | 4 copies | | |
| | | | | | | | - | - | *EGFR* amplification | 3 copies | | |
| | | | | | | | - | - | *CDK6* amplification | 3 copies | | |
| | | | | | | | - | - | *CCND3* amplification | 3 copies | | |
| | | | | | | | - | - | *CCND1* amplification | 4 copies | | |
| #14 | F | N | IV | + | Y | ICB | *BRAF* p.Val600Glu | 2.4 | *BRAF* p.Val600Glu | 43.8 | 51 | 21 |
| | | | | | | | *PIK3CA* p.Asn345Lys | 1.2 | - | - | | |
| | | | | | | | - | - | *CCND1* amplification | 3 copies | | |
| | | | | | | | - | - | *EGFR* amplification | 3 copies | | |
| | | | | | | | - | - | *FGFR3* amplification | 4 copies | | |
| #19 | M | N | IV | + | Y | BRAF+MEKi | BRAF p.Val600Glu | 0.7 | *BRAF* p.Val600Glu | 36.4 | 83 | 56 |
| | | | | | | | NRAS p.Gln61Arg | 0.2 | - | - | | |
| | | | | | | | - | - | *FGFR2* deletion | 1 copy | | |
| #60_T1 | F | N | IIIB | + | N | BRAF+MEKi adjuvant | - | - | *BRAF* p.Val600Glu | 76.0 | 28 | 569 |
| #60_T2 | F | N | IIIB | + | N | BRAF+MEKi adjuvant | - | - | *BRAF* p.Val600Glu | 76.0 | 329 | 871 |
| #60_T3 | F | N | IIIB | + | N | BRAF+MEKi adjuvant | - | - | *BRAF* p.Val600Glu | 76.0 | 656 | 1198 |
| #62_T1 | M | N | IV | + | N | - | *BRAF* p.Val600Glu | 12.3 | *BRAF* p.Val600Glu | 22.8 | 0 | 0 |

| | | | | | | | | | | | | |
|---|---|---|---|---|---|---|---|---|---|---|---|---|
| | | | | | | | *PIK3CA* p.Glu545Lys | 0.4 | - | - | | |
| #62_T2 | M | N | IV | + | N | BRAF+MEKi | *BRAF* p.Val600Glu | 39.2 | *BRAF* p.Val600Glu | 17.0 | -7 | 110 |
| | | | | | | | *GNA11* p.Gln209Leu | 15.7 | - | - | | |
| | | | | | | | PIK3CA p.Glu545Lys | 1.3 | - | - | | |
| #7 | F | Y | IV | - | N | ICB | *BRAF* p.Val600Glu | 0.8 | - | - | 825 | 1002 |
| | | | | | | | *KIT* p. Lys642Glu | 0.5 | - | - | | |
| | | | | | | | *MET* p.Arg988Cys 5 | 51.8 | - | - | | |
| | | | | | | | - | - | *IDH1* p.Arg132Cys | 23.0 | | |
| | | | | | | | - | - | *CCND3* amplification | 3 copies | | |
| #57 | M | N | IV | - | Y | ICB adjuvant and Imatinib | *BRAF* p.Gly469Ala | 46.7 | *BRAF* p.Gly469Ala | 58.7 | -6 | 441 |
| | | | | | | | *KIT* p.Lys642Glu | 34.6 | *KIT* p.Lys642Glu | 47.0 | | |
| | | | | | | | *EGFR* amplifications | 1.32 copies | *EGFR* amplifications | 3 copies | | |
| | | | | | | | *CDK6* amplifications | 1.42 copies | *CDK6* amplifications | 3 copies | | |
| | | | | | | | *MET* amplifications | 1.31 copies | - | - | | |

*Abbreviations: R: Response; Y: yes; N: no; AF: Allele Frequency; ICB: Immunological Checkpoint Blocking; BRAF+MEKi: BRAF and MEK inhibitors.*

**Supplementary Table S11.** Clinical characteristics of the enrolled patients.

| Patient ID | *BRAF* V600 Status | Adjuvant therapy | 1st line therapy | RECIST BOR 1st line therapy | PFS 1st line therapy | 2nd line therapy | RECIST BOR 2nd line therapy | PFS 2nd line therapy | iAEs (G3-G5) | CLINICAL BENEFIT | | |
|---|---|---|---|---|---|---|---|---|---|---|---|---|
| | | | | | | | | | | Adjuvant | TT | ICI |
| **#1** | + | No | BRAF+MEKi | SD | 7,53 | Anti-PD-1 | PD | 0,93 | No | NA | 0 | 0 |
| **#2** | + | No | BRAF+MEKi | SD | 11,10 | Anti-PD-1 | PD | 2,93 | No | NA | 1 | 0 |
| **#3** | + | No | BRAF+MEKi | PD | 3,07 | No | NA | NA | No | NA | 0 | NA |

| | | | | | | | | | | | | |
|---|---|---|---|---|---|---|---|---|---|---|---|---|
| **#8** | + | No | BRAF+MEKi | SD | 8,17 | No | NA | NA | No | NA | 0 | NA |
| **#10** | + | No | BRAF+MEKi | SD | 5,73 | Anti-PD-1 | PR | 9,07 | No | NA | 0 | 1 |
| **#14** | + | No | Anti-CTLA-4 + Anti-PD-1 | PD | 1,37 | BRAF+MEKi | PD | 4,73 | Colitis (G4) | NA | 0 | 0 |

| | | | | | | | | | | | | |
|---|---|---|---|---|---|---|---|---|---|---|---|---|
| #**17** | + | No | Anti-PD-1 | SD | 11,20 | No | NA | NA | Pneumonia (G1) | NA | NA | 1 |
| #**19** | + | No | BRAF+MEKi | SD | 4,20 | Anti-PD-1 | PD | 0,37 | No | NA | 0 | 0 |
| #**26** | + | No | BRAF+MEKi | PR | 13,07 | BRAF+MEKi | PD | 5,43 | No | NA | 1 | NA |

| | | | | | | | | | | | | |
|---|---|---|---|---|---|---|---|---|---|---|---|---|
| **#30** | + | No | Anti-PD-1 | PR | 7,93 | No | NA | NA | Vitiligo | NA | NA | 1 |
| **#37** | + | No | BRAF+MEKi | PR | 20,33 | Anti-PD-1 | PD | 1,46 | No | NA | 1 | 0 |
| **#39** | + | No | BRAF+MEKi | PR | 24,97 | Anti-PD-1 | SD | 7,33 | No | NA | 1 | 1 |

| | | | | | | | | | | | | |
|---|---|---|---|---|---|---|---|---|---|---|---|---|
| **#40** | + | No | BRAF+MEKi | PR | 19,63 | Anti-PD-1 | SD | 12,2 | No | NA | 1 | 1 |
| **#42** | + | No | BRAF+MEKi | PR | 20,10 | No | NA | NA | No | NA | 1 | NA |
| **#43** | + | Adjuvant with BRAF+MEKi | BRAF+MEKi | PR | 4,90 | No | NA | NA | No | 0 | 0 | NA |

| | | | | | | | | | | | | |
|---|---|---|---|---|---|---|---|---|---|---|---|---|
| **#50** | + | No | Anti-PD-1 | PD | 1,00 | BRAF+MEKi | PR | 2,96 | No | 0 | 0 | 0 |
| **#56** | + | Adjuvant with BRAF+MEKi | Anti-PD-1 | PD | 2,00 | Chemotherapy | PD | 1,93 | No | 0 | NA | 0 |
| **#60** | + | Adjuvant with BRAF+MEKi | No | NA | NA | No | NA | NA | No | 0 | NA | NA |

| | | | | | | | | | | | | |
|---|---|---|---|---|---|---|---|---|---|---|---|---|
| | | Adjuvant with anti-PD-1 | | | | | | | | 0 | | |
| #**62** | + | No | BRAF+MEKi | PR | 3,60 | Anti-PD-1 | PD | 1,2 | No | NA | 0 | 0 |
| #**4** | - | No | Anti-CTLA-4 | PD | 5,27 | Anti-PD-1 | PR | 13,1 | Thyroiditis and hypophysitis | NA | NA | 1 |

| | | | | | | | | | | | | |
|---|---|---|---|---|---|---|---|---|---|---|---|---|
| **#6** | - | No | Anti-PD-1 | SD | 10,30 | Anti-CTLA-4 | PR | 6,76 | No | NA | NA | 1 |
| **#7** | - | No | Anti-PD-1 | PR | 64,20 | No | NA | NA | Bullous pemphigoid | NA | NA | 1 |
| **#18** | - | No | Anti-PD-1 | PD | 5,90 | Chemotherapy | PD | 0,77 | No | NA | NA | 0 |

| | | | | | | | | | | | | |
|---|---|---|---|---|---|---|---|---|---|---|---|---|
| **#20** | - | No | Anti-PD-1 | SD | 3,73 | Anti-PD-1 | CR | 13,06 | No | NA | NA | 1 |
| **#21** | - | Anti-PD-1 +/- anti-CTLA-4 | No | NA | NA | No | NA | NA | Vitiligo | 1 | NA | NA |
| **#32** | - | No | Anti-PD-1 | PD | 9,33 | Anti CTLA-4 | PR | 13,86 | Vitiligo | NA | NA | 1 |

| | | | | | | | | | | | | |
|---|---|---|---|---|---|---|---|---|---|---|---|---|
| **#34** | - | No | Anti-PD-1 | PD | 2,80 | No | NA | NA | No | NA | NA | 0 |
| **#38** | - | No | Anti-PD-1 | PD | 4,57 | Anti-CTLA-4 | SD | 10,86 | Amylase and lipase | NA | NA | 1 |
| **#45** | - | No | Anti-PD-1 | PD | 1,53 | No | NA | NA | No | NA | NA | 0 |

| | | | | | | | | | | | | |
|---|---|---|---|---|---|---|---|---|---|---|---|---|
| **#46** | - | No | Anti-PD-1 | PR | 29,43 | No | NONo | NA | Hypothyroidism and arthritis | NA | NA | 1 |
| **#47** | - | Adjuvant with anti-PD-1 | c-Kit-i | PR | 6,33 | Anti-CTLA-4 | PD | 1,1 | No | 0 | 0 | 0 |
| **#54** | - | Adjuvant with anti-PD-1 | No | NA | NA | NA | NA | NA | Pancreatitis | 0 | NA | NA |

| | | | | | | | | | | | | |
|---|---|---|---|---|---|---|---|---|---|---|---|---|
| **#55** | - | Adjuvant with anti-PD-1 | Anti-CTLA-4 | PD | 3,80 | NA | NA | NA | Diarrhea | 0 | NA | 0 |
| **#57** | - | Adjuvant with anti-PD-1 | c-Kit-i | PR | 8,03 | Chemotherapy | PD | 1,8 | No | 0 | 0 | 0 |
| **#59** | - | Adjuvant with anti-PD-1 | No | NA | NA | NA | NA | NA | No | 1 | NA | NA |

| | | | | | | | | | | | | |
|---|---|---|---|---|---|---|---|---|---|---|---|---|
| **#63** | - | Adjuvant with anti-PD-1 | Anti-PD-1 | SD | 4,47 | NA | NA | NA | No | 0 | NA | 1 |

Anti-PD-1: nivolumab, pembrolizumab; Anti-CTLA-4: ipilimumab; BRAF+MEKi: vemurafenib + cobimetinib, dabrafenib + trametinib, encorafenib + binimetinib; c-KITi: imatinib.

*Abbreviations:* PD: NA: Not Available; SD: Stable Disease; PR: Partial Response; CR: Complete Response; PD: Disease Progression: ICB: Immunological Checkpoint Blocking; BOR: Best Overall Response; PFS: Progression Free Survival: iAEs. immuno-related Adverse Events; O: No clinical Benefit; 1: Clinical Benefit.
