# Supplementary figures and images for: Whole-Exome Sequencing and cfDNA Analysis Uncover Genetic Determinants of Melanoma Therapy Response in a Real-World Setting
Source: Int J Mol Sci. 2023 Feb 21;24(5):4302. doi: 10.3390/ijms24054302 (PMC10002464; doi:10.3390/ijms24054302)

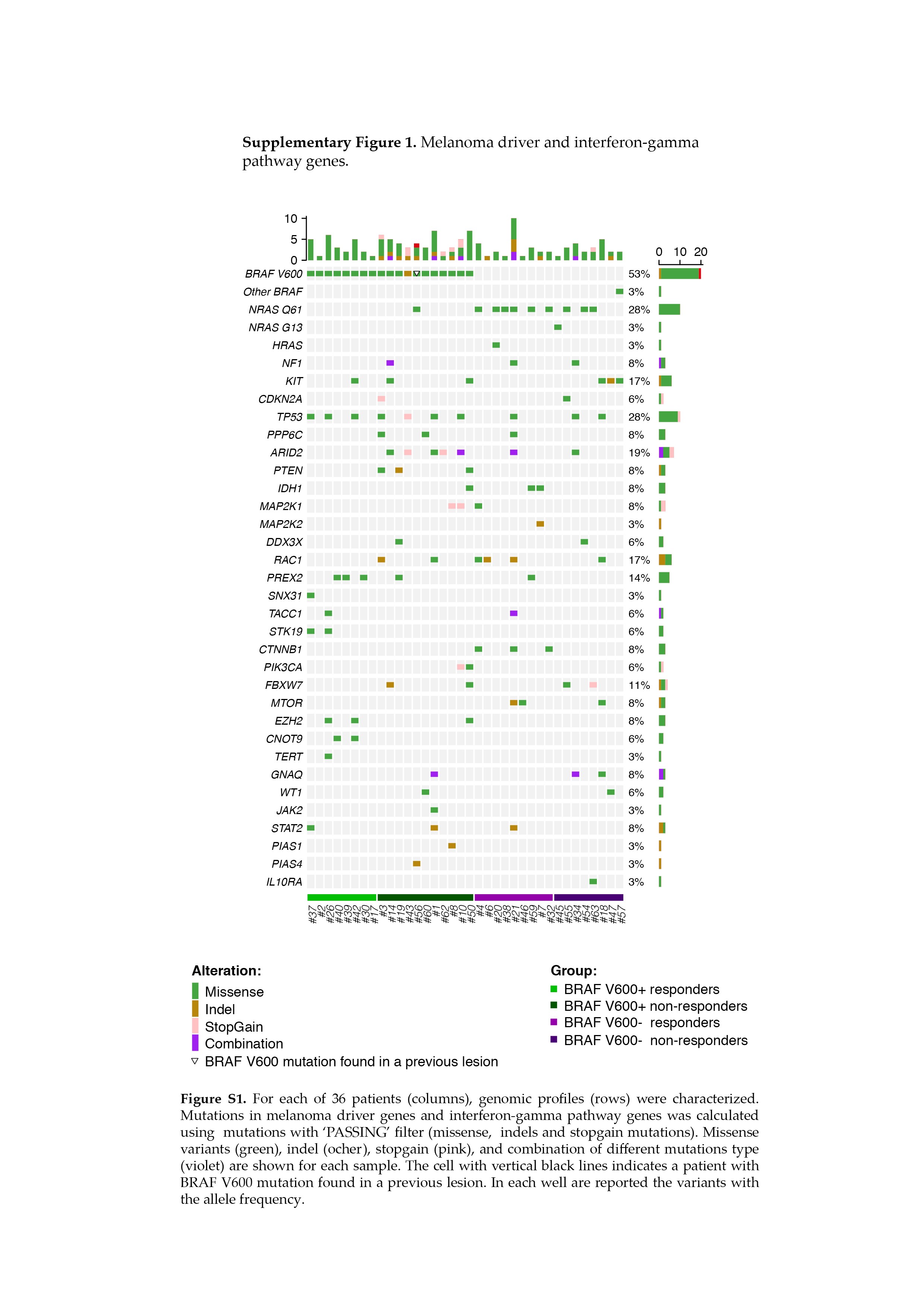

Supplement: Supplementary file 1 [file ijms-24-04302-s001.zip › Supplementary Figure 1_proof.jpg]

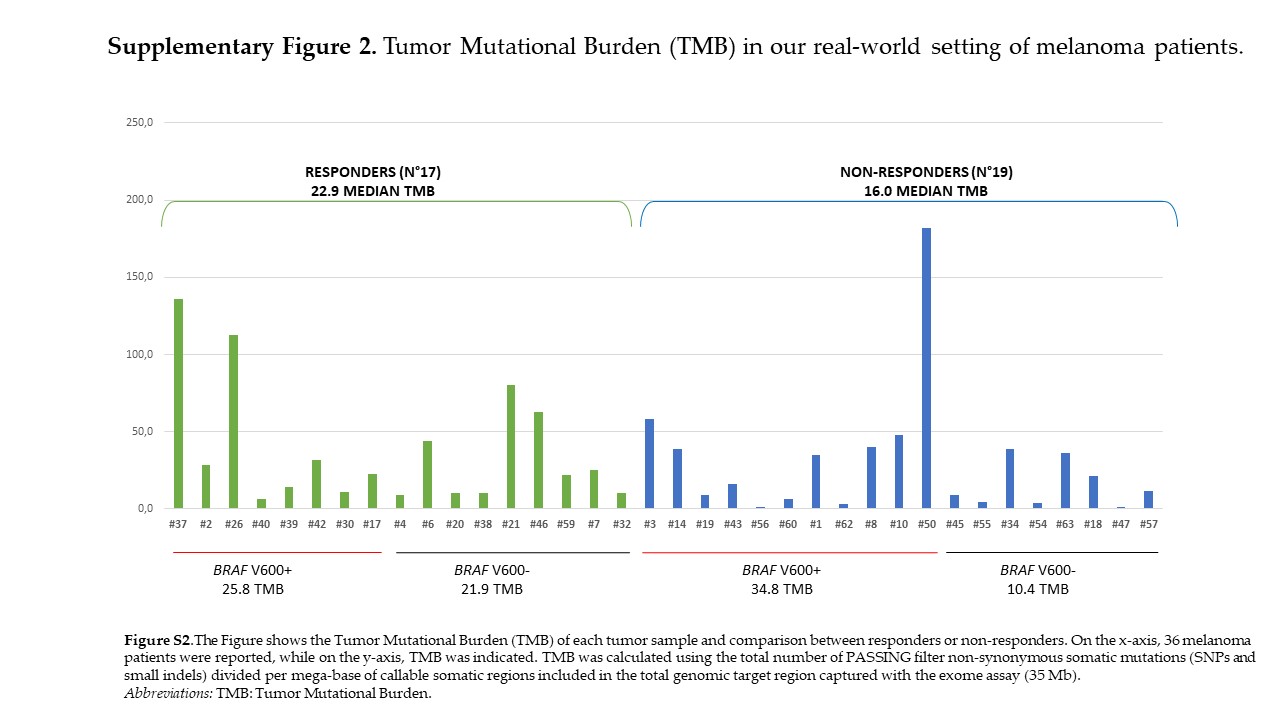

Supplement: Supplementary file 1 [file ijms-24-04302-s001.zip › Supplementary Figure 2_proof.jpg]

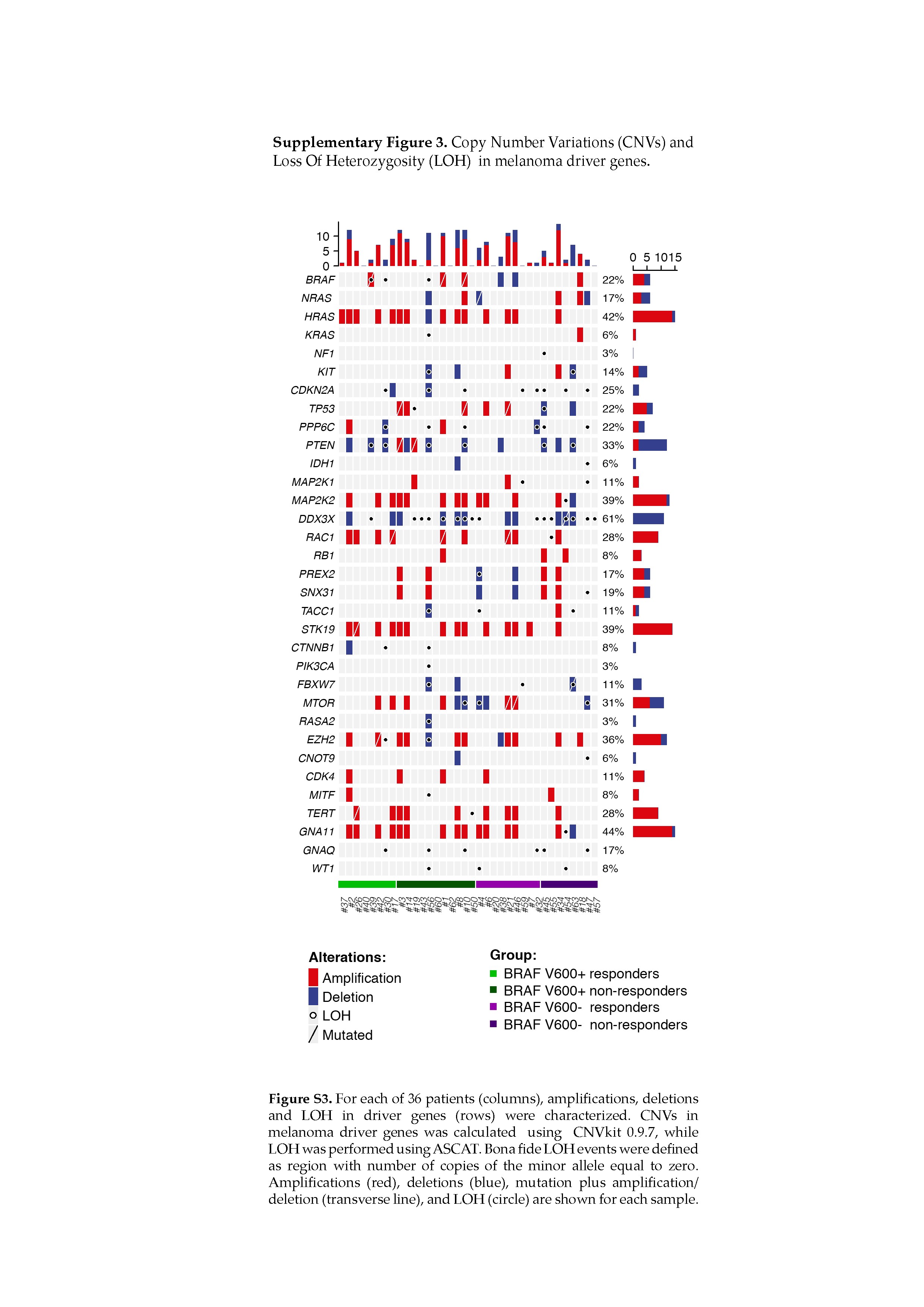

Supplement: Supplementary file 1 [file ijms-24-04302-s001.zip › Supplementary Figure 3_proof.jpg]

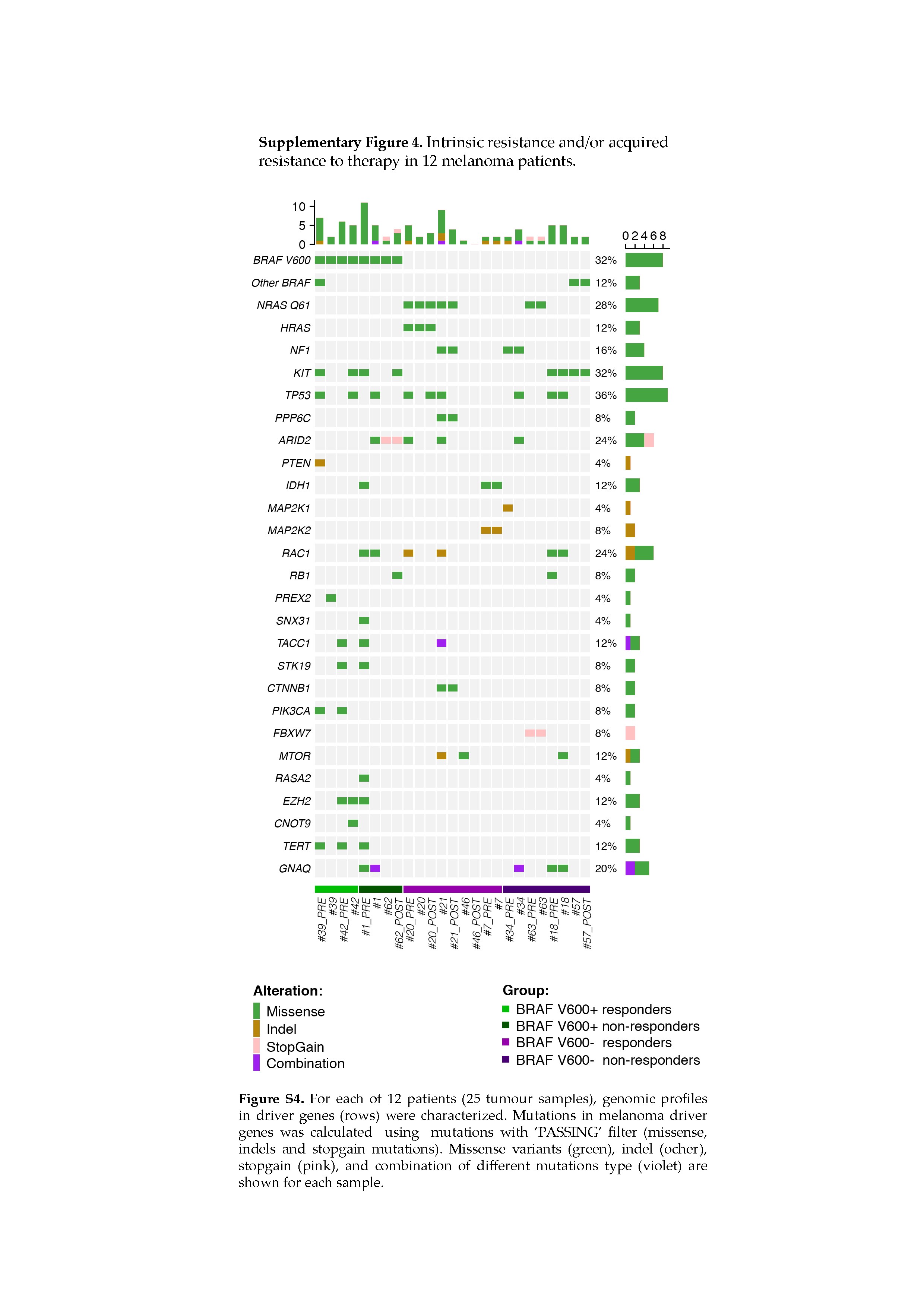

Supplement: Supplementary file 1 [file ijms-24-04302-s001.zip › Supplementary Figure 4_proof.jpg]

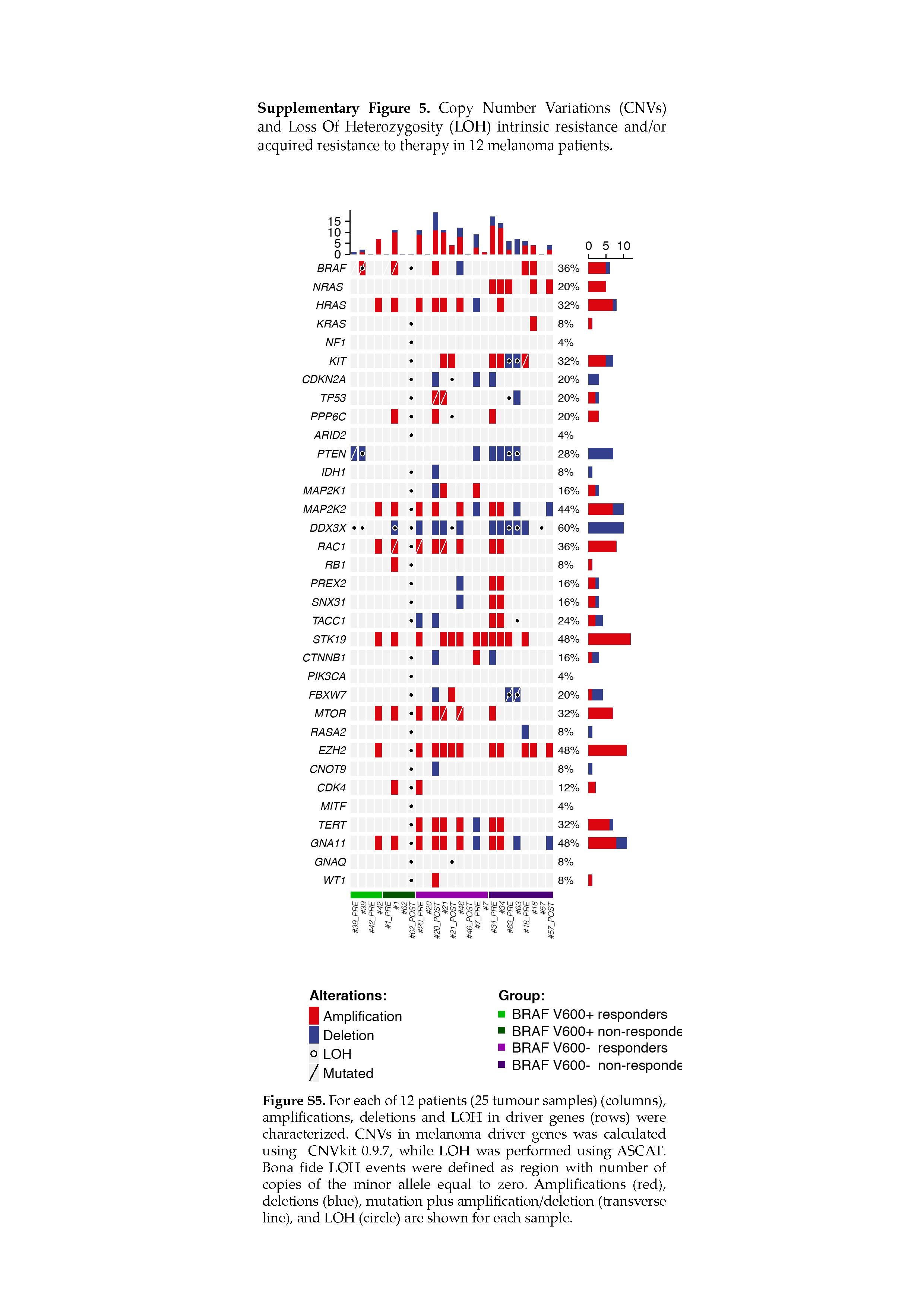

Supplement: Supplementary file 1 [file ijms-24-04302-s001.zip › Supplementary figure 5_proof.jpg]

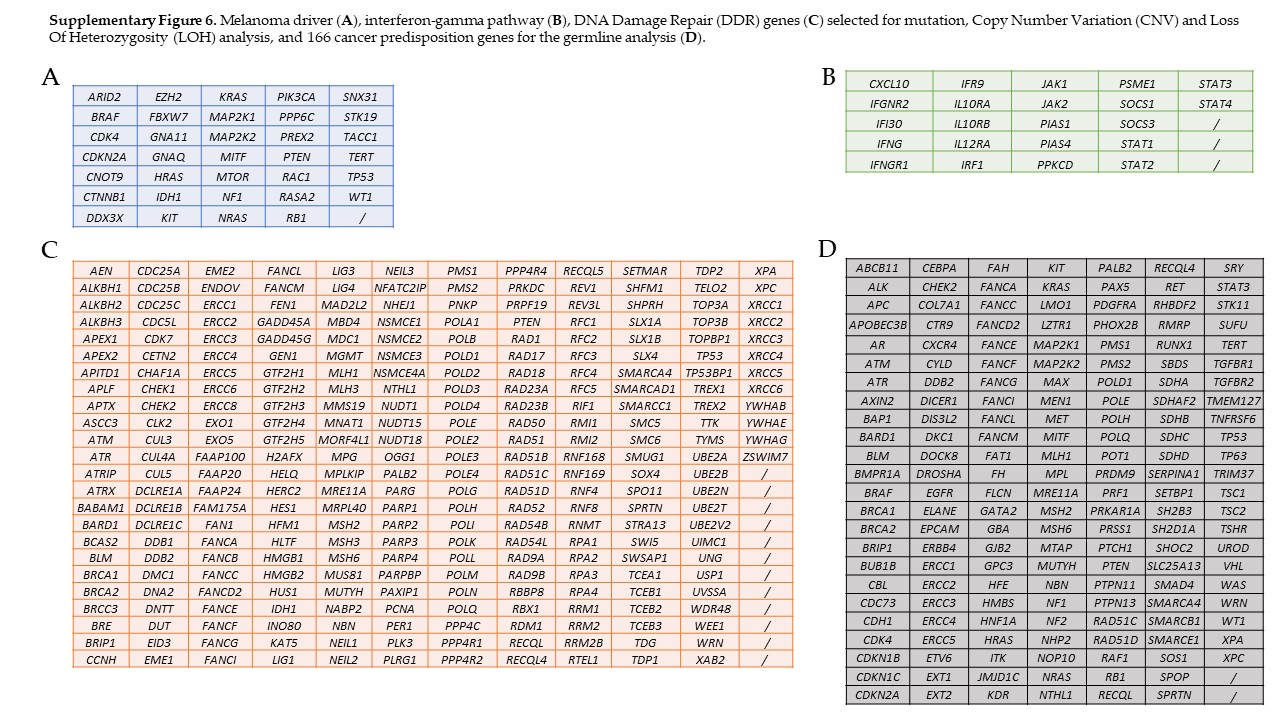

Supplement: Supplementary file 1 [file ijms-24-04302-s001.zip › Supplementary Figure 6_proof.jpg]
